# Supplementary material for: Overcoming the low reactivity of biobased, secondary diols in polyester synthesis
Source: Nat Commun. 2022 Nov 30;13:7370. doi: 10.1038/s41467-022-34840-2 (PMC9712608; doi:10.1038/s41467-022-34840-2)
Supplement: Supplementary file 1 — Supplementary Information [file 41467_2022_34840_MOESM1_ESM.pdf]

Supplementary Information

Overcoming the low reactivity of biobased, secondary diols in polyester synthesis

Daniel H. Weinland[1], Kevin van der Maas[1], Yue Wang[1], Bruno Bottega Pergher[1], Robert-Jan van Putten[1,2], Bing Wang[2], Gert-Jan M. Gruter[1,2] *

Affiliations

[1] *Van't Hoff Institute of Molecular Sciences, University of Amsterdam, P.O. Box 94720, 1090GS Amsterdam, The Netherlands*

[2] *Avantium Chemicals BV, Zekeringstraat 29, 1014BV Amsterdam, The Netherlands*

* *Corresponding author*. E-mail address*:* g.j.m.gruter@uva.nl

Table of Contents

Supplementary Methods 4
Evolution of reactants during esterification between isosorbide, succinic acid and *p*-cresol 4
Relevant end groups after 5 h esterification between isosorbide, succinic acid and *p*-cresol 5
Calculation of alcohol to ester end group ratio after esterification 6
Calculation of unreacted isosorbide 6
Comparison of ^{1}H NMR spectra of reaction mixtures of isosorbide polyesters after esterification 7
Comparison of ^{1}H NMR spectra of reaction mixtures of isomannide polyesters after esterification 8
Unreacted aryl alcohol in polymer products 9
Additional characterization of poly(isosorbide succinate) (PIsSu) 10
Additional characterization of poly(isosorbide glutarate) (PIsGlu) 12
Additional characterization of poly(isosorbide adipate) (PIsAd) 13
Additional characterization of poly(isosorbide-1,4-cyclohexanedicarboxylate) (PIsCyc) 14
Additional characterization of poly(isosorbide diglycolate) (PIsDga) 16
Additional characterization of poly(isosorbide thiodiglycolate) (PIsThd) 19
Additional characterization of poly(isomannide succinate) (PImSu) 20
Additional characterization of poly(isomannide glutarate) (PImGlu) 22
Additional characterization of poly(isomannide adipate) (PImAd) 23
Additional characterization of poly(isomannide-1,4-cyclohexanedicarboxylate) (PImCyc). 24
Additional characterization of poly(isomannide diglycolate) (PImDga) 27
DSC traces of isosorbide-based polyesters 30
DSC traces of isomannide-based polyesters 30
Thermal stability of isosorbide- and isomannide-based polyesters 32
O_2 and H_2O permeability curves for isosorbide-based polyesters 33
Stress-strain graphs of isosorbide-based polyesters 34
Comparison of 100 mL glass reactor with 2 L steel reactor 35
Collection of volatiles during esterification of poly(isosorbide succinate) in 2 L autoclave . 36
Ring-opening hydration of isosorbide to 1,4-sorbitan 36
Calculation of mol% (respective total isosorbide units) of 1,4-sorbitan during esterification in 2 L autoclave 38
Temperature, torque and pressure evolution during poly(isosorbide succinate) synthesis in 2 L autoclave 39
Poly(isosorbide succinate) from 2 L autoclave 39
Soil burial degradability of poly(isosorbide succinate) 40

Hydrolytic degradability of poly(isosorbide succinate) 40
Supplementary Tables 41
Synthesis conditions for isosorbide-and isomannide-based polyesters 41
Alcohol to ester end group ratios and mol% of unreacted diol monomers of all polyester compositions after esterification 42
$T_{5\%d}$ values of polyester compositions 43
Conditions used for compression moulding of polymer films and average thicknesses 43
Conditions used for injection moulding of polymer tensile bars 44
Influence of injection moulding on PIsSu molecular weight 44
Comparison of mechanical properties of PIsSu synthesized in glass reactor and 2 L autoclave 44
Comparison of barrier properties of PIsSu synthesized in glass reactor and 2 L autoclave 45

Supplementary Methods

Evolution of reactants during esterification between isosorbide, succinic acid and *p*-cresol

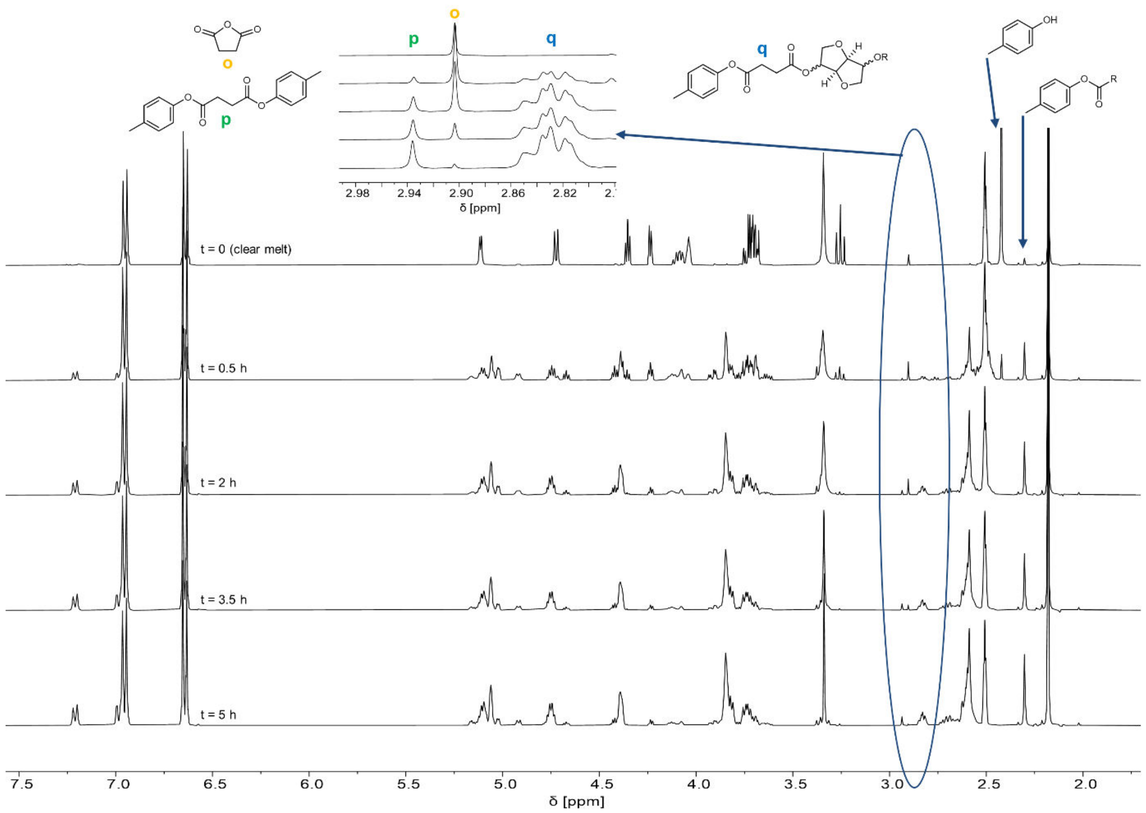

Supplementary Figure 1. ^{1}H NMR spectra recorded during esterification between isosorbide, succinic acid and *p*-cresol at t = 0 to t = 5 h. Highlighted are the peaks between 2.8 and 3.0 ppm, which indicate the presence of succinic anhydride, bis(*p*-cresyl)succinate and *mono*-*p*-cresyl succinate esters.

Relevant end groups after 5 h esterification between isosorbide, succinic acid and *p*-cresol

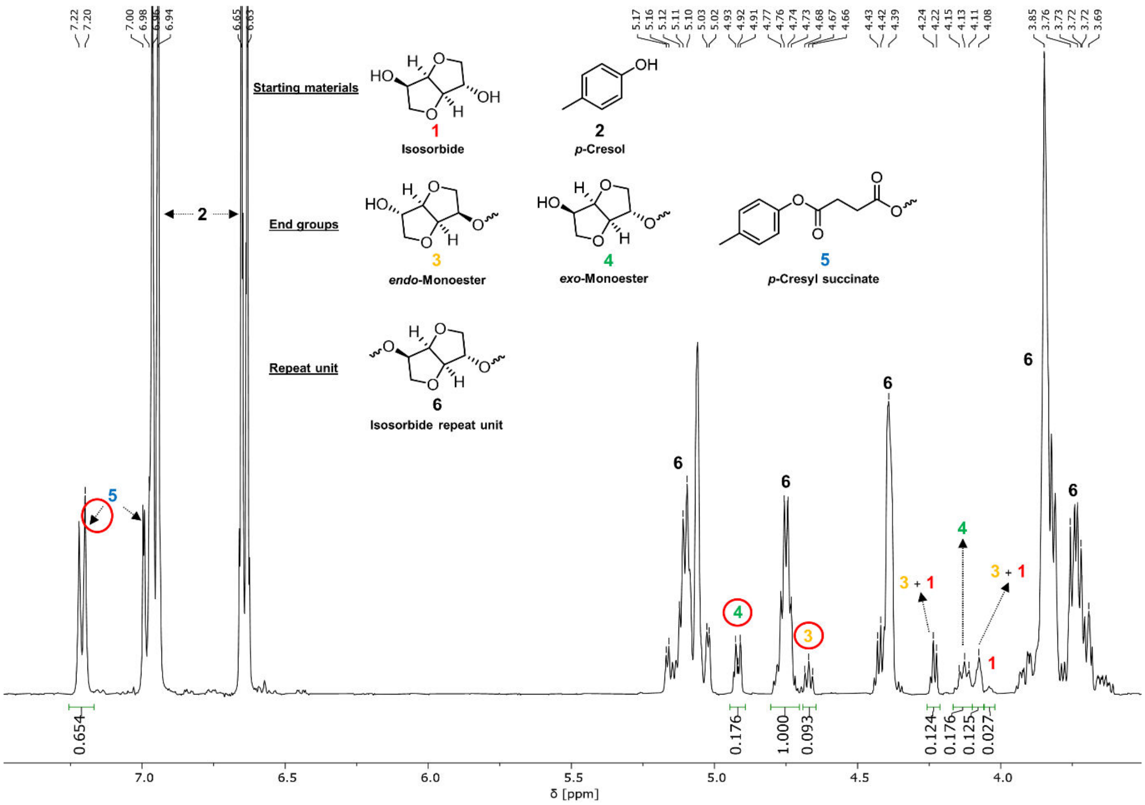

Supplementary Figure 2. ^{1}H NMR spectrum of the reaction mixture after 5 h esterification. The relevant peaks for the calculation of the alcohol to cresyl succinate end group ratio are highlighted. Isosorbide assignments were done on the basis of integral ratios, considering the lower reactivity of *endo*-OH. For a complete assignment of the isosorbide related end group signals, see Noordover *et al.*[1]

Calculation of alcohol to ester end group ratio after esterification

To estimate the conversion of esterification reactions, the alcohol to ester end group ratios in reactions with isosorbide were calculated as follows.

$$Ratio\ (alcohol\ to\ ester\ end\ groups) = \frac{A(endo-isosorbide\ monoester, 4.67\ ppm, 1H) + A(exo-isosorbide\ monoester, 4.93\ ppm, 1H)}{\frac{A(p-cresyl\ succinate\ ester, 7.22\ ppm, 2H)}{2}}$$

For an assignment of the relevant end groups, see Supplementary Figure 2. Successful polyester syntheses typically yielded an alcohol to ester end group ratio after esterification between 0.85 and 0.95. The *p*-cresyl succinate peak at 7.22 ppm was used for calculations of the end group ratios despite a slight overestimation due to the presence of bis(*p*-cresyl) succinate (see Supplementary Figure 1). This is due to the easy identification of the *p*-cresyl esters around 7.2 ppm, independent of the diacid moiety used (see Supplementary Figure 3, Supplementary Figure 4 for ^{1}H NMR spectra of esterification mixtures of all described polyester compositions).

Similarly, calculations were carried out for isomannide-based polyesters. The equivalence of isomannide's two *endo*-OH groups resulted in only one signal for the respective monoesters.

$$Ratio\ (alcohol\ to\ ester\ end\ groups) = \frac{A(isomannide\ monoester, 4.90\ ppm, 1H)}{\frac{A(p-cresyl\ succinate\ ester, 7.22\ ppm, 2H)}{2}}$$

For an overview of alcohol to ester end group ratios after esterification for all synthesized polyesters, see Supplementary Table 2.

Calculation of unreacted isosorbide

The amount of total unreacted isosorbide was calculated as follows:

$$Total\ unreacted\ isosorbide\ (respective\ total\ mol\%\ of\ isosorbide) = \frac{A(unreacted\ IS, 4.03\ ppm, 1H)}{A(endo-IS\ monoester, 4.67\ ppm, 1H) + A(exo-IS\ monoester, 4.93\ ppm, 1H) + A\ (IS\ repeat\ unit, 4.74\ ppm, 1H) + A(unreacted\ IS, 4.03\ ppm, 1H)} * 100$$

Comparison of ^{1}H NMR spectra of reaction mixtures of isosorbide polyesters after esterification

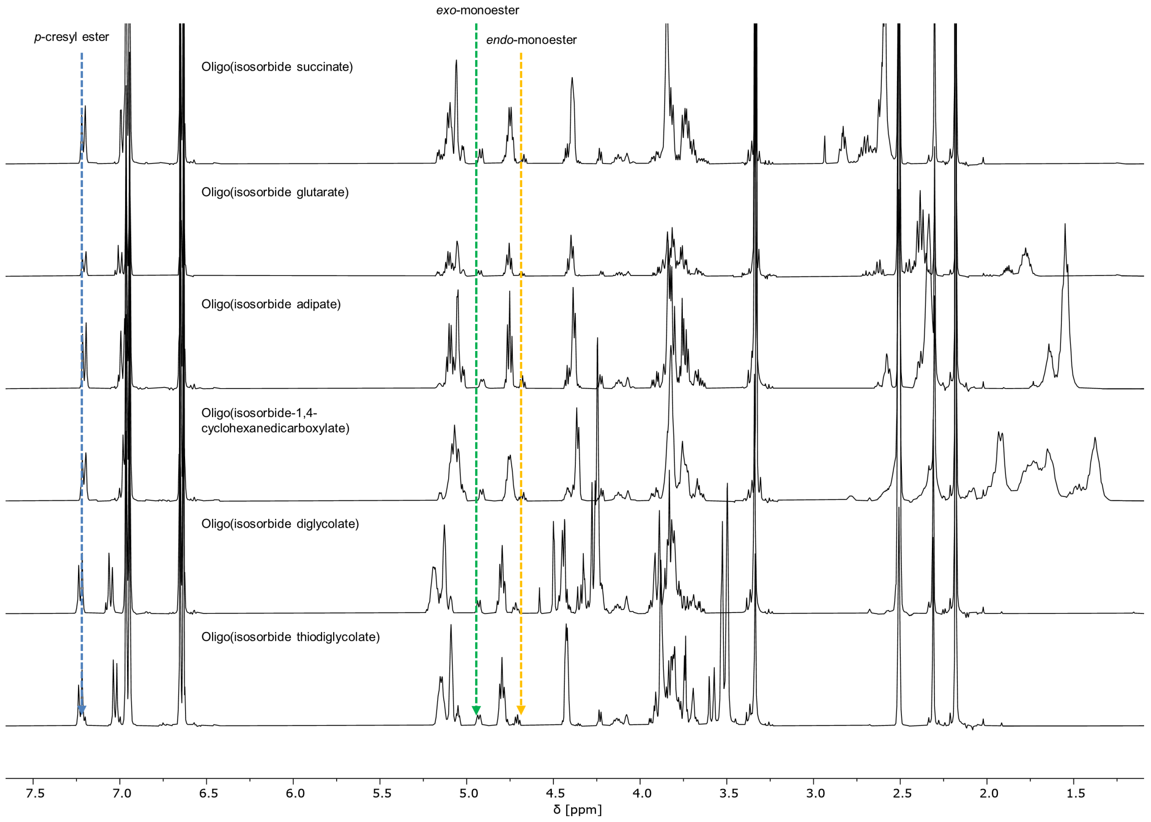

Supplementary Figure 3. Comparison of ^{1}H NMR spectra taken after complete esterification of isosorbide-based polyesters. End group signals relevant for calculation of alcohol to ester end group ratios are highlighted.

Comparison of ^{1}H NMR spectra of reaction mixtures of isomannide polyesters after esterification

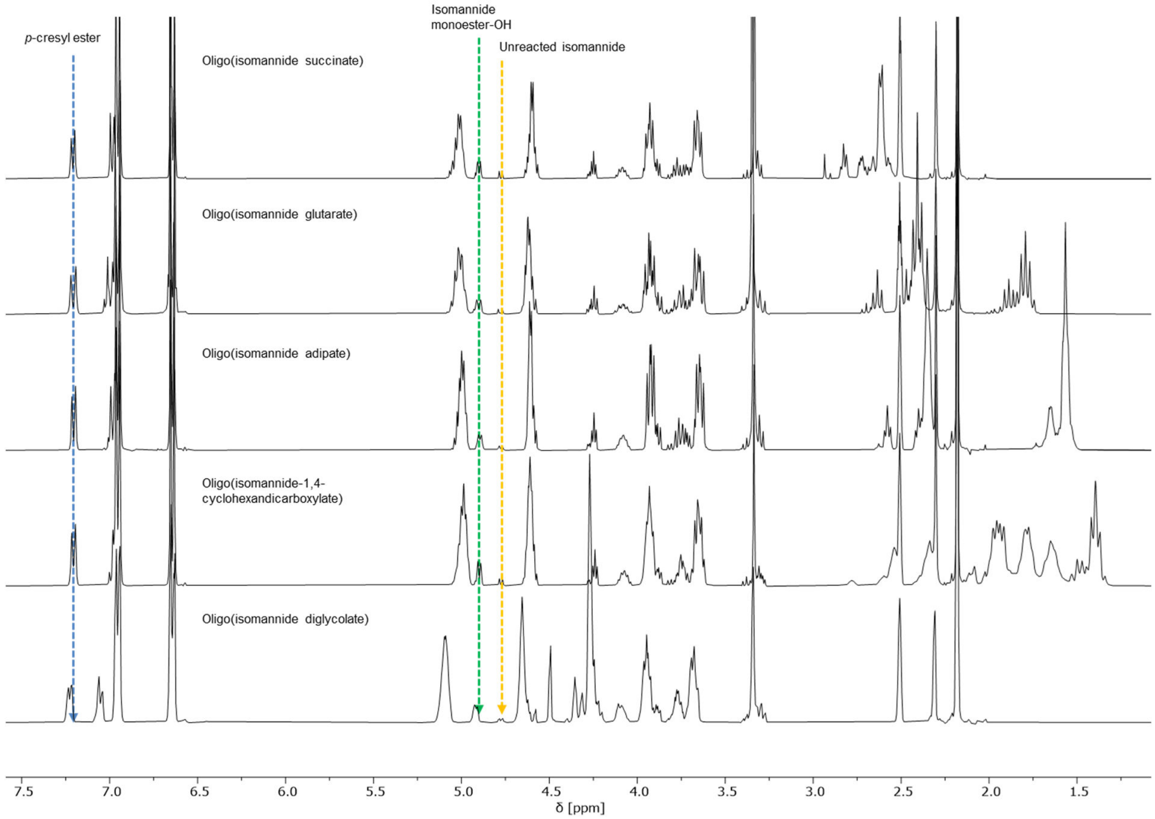

Supplementary Figure 4. Comparison of ^{1}H NMR spectra taken after complete esterification of isomannide-based polyesters. End group signals relevant for calculation of alcohol to ester end group ratios are highlighted.

Unreacted aryl alcohol in polymer products

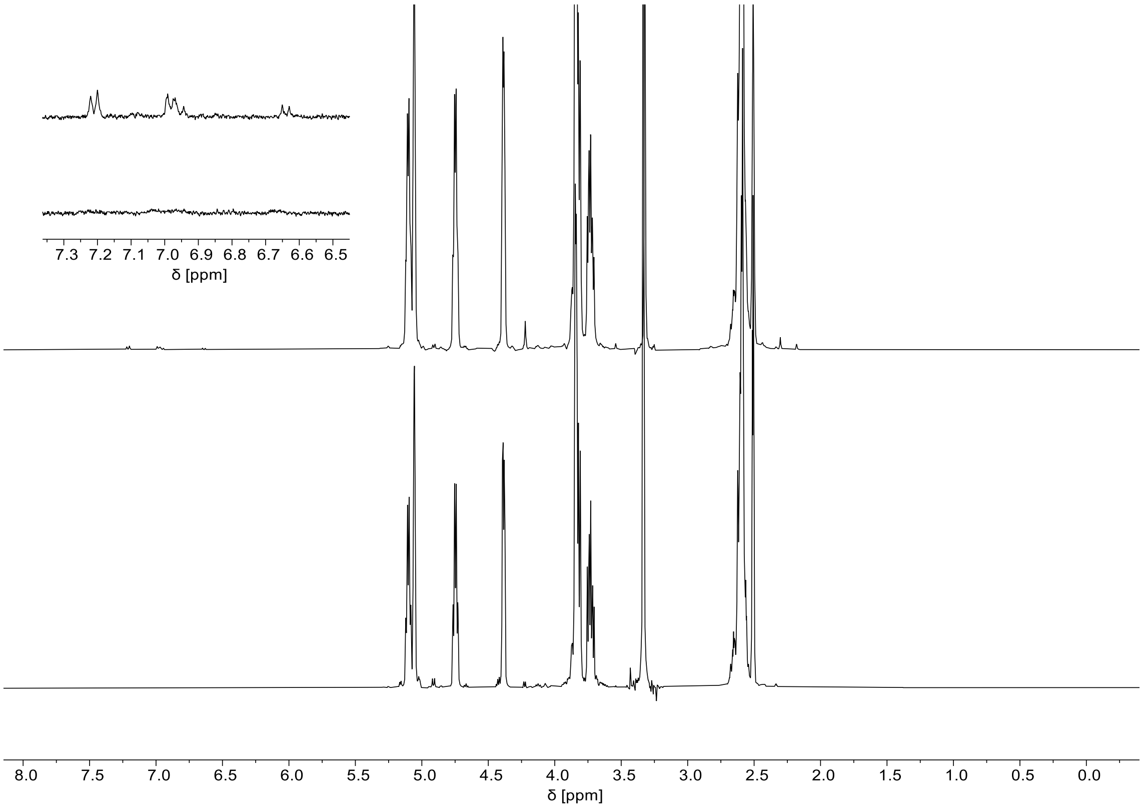

Supplementary Figure 5. Comparison of ^{1}H NMR spectra of poly(isosorbide succinate) synthesized without (top) and with (bottom, 1.5 mol% respective succinic acid) an isosorbide excess. The signal at 6.64 ppm corresponds to unreacted *p*-cresol. An excess of diols reduces the amount of free *p*-cresol in the final polymer below the detection limit of ^{1}H NMR spectroscopy.

Additional characterization of poly(isosorbide succinate) (PIsSu)

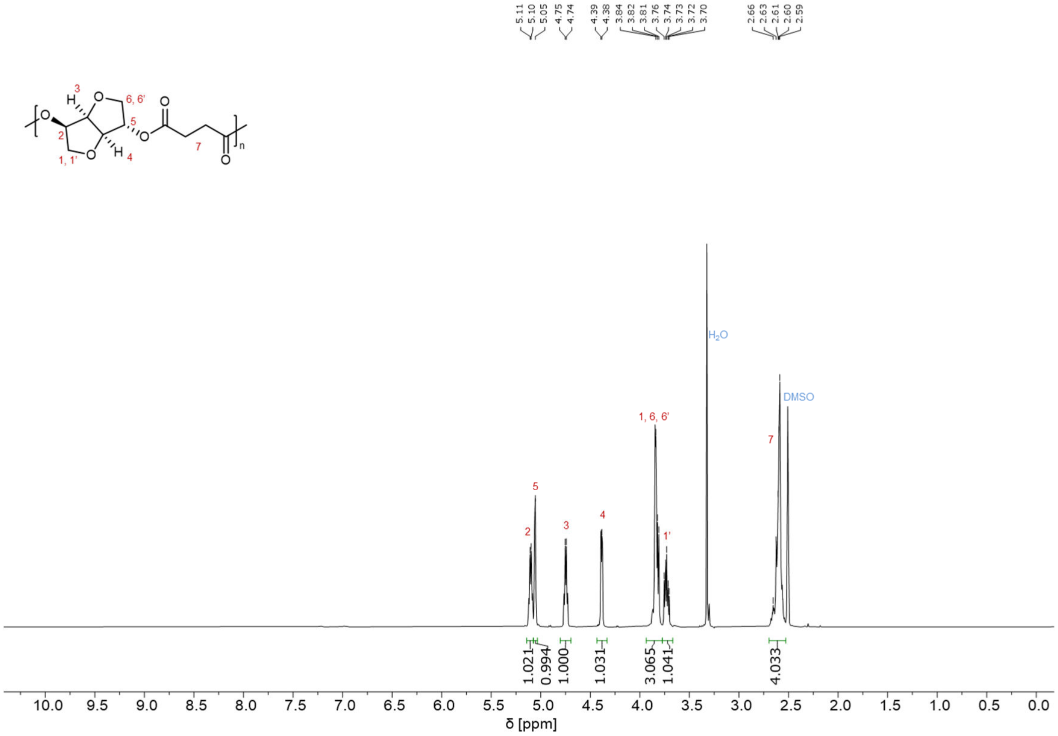

Supplementary Figure 6. ^{1}H NMR spectrum (298 K, 500 MHz) of poly(isosorbide succinate) in DMSO-d6.

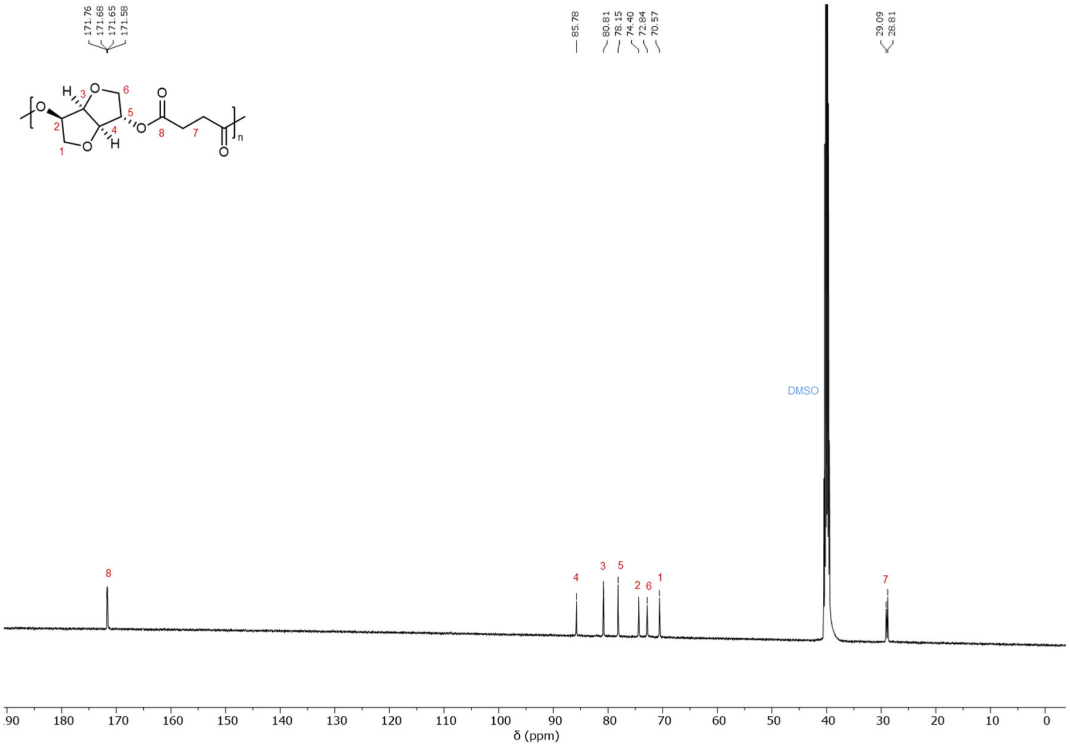

Supplementary Figure 7. ^{13}C NMR spectrum (298 K, 125 MHz) of poly(isosorbide succinate) in DMSO-d6.

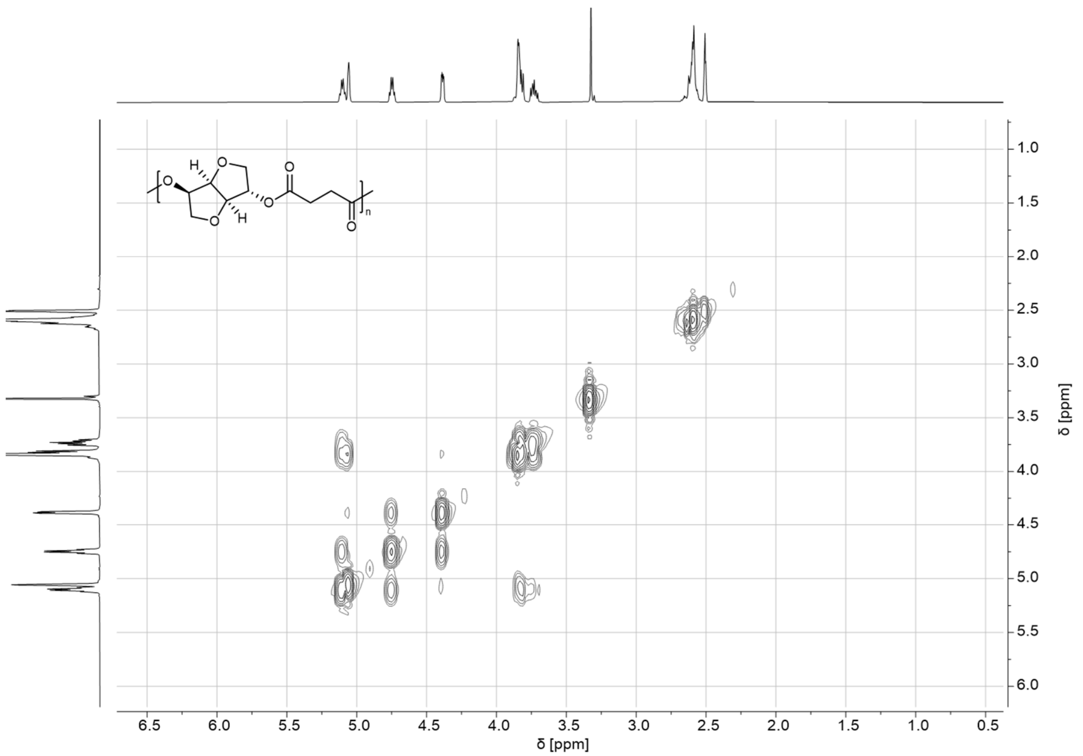

Supplementary Figure 8. ^{1}H-^{1}H COSY NMR spectrum (298 K, 500 MHz) of poly(isosorbide succinate) in DMSO-d6.

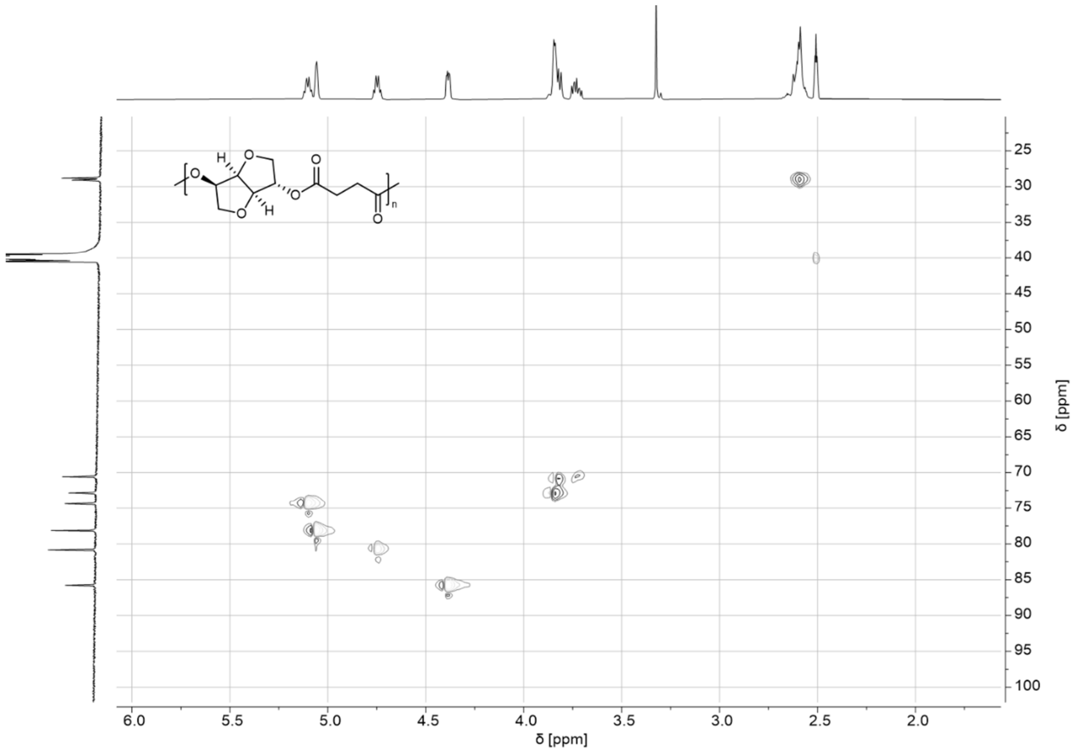

Supplementary Figure 9. ^{1}H-^{13}C HSQC NMR spectrum (298 K, 500/125 MHz) of poly(isosorbide succinate) in DMSO-d6.

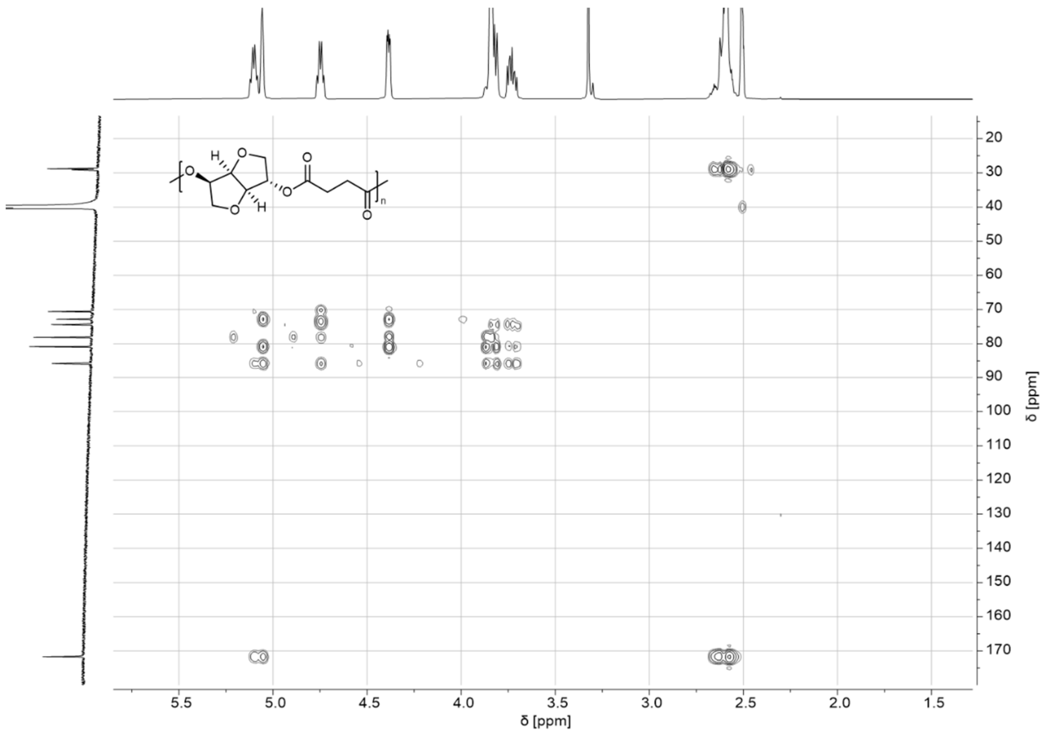

Supplementary Figure 10. ^1H-^{13}C HMBC NMR spectrum (298 K, 500/125 MHz) of poly(isosorbide succinate) in DMSO-d6.

Additional characterization of poly(isosorbide glutarate) (PIsGlu)

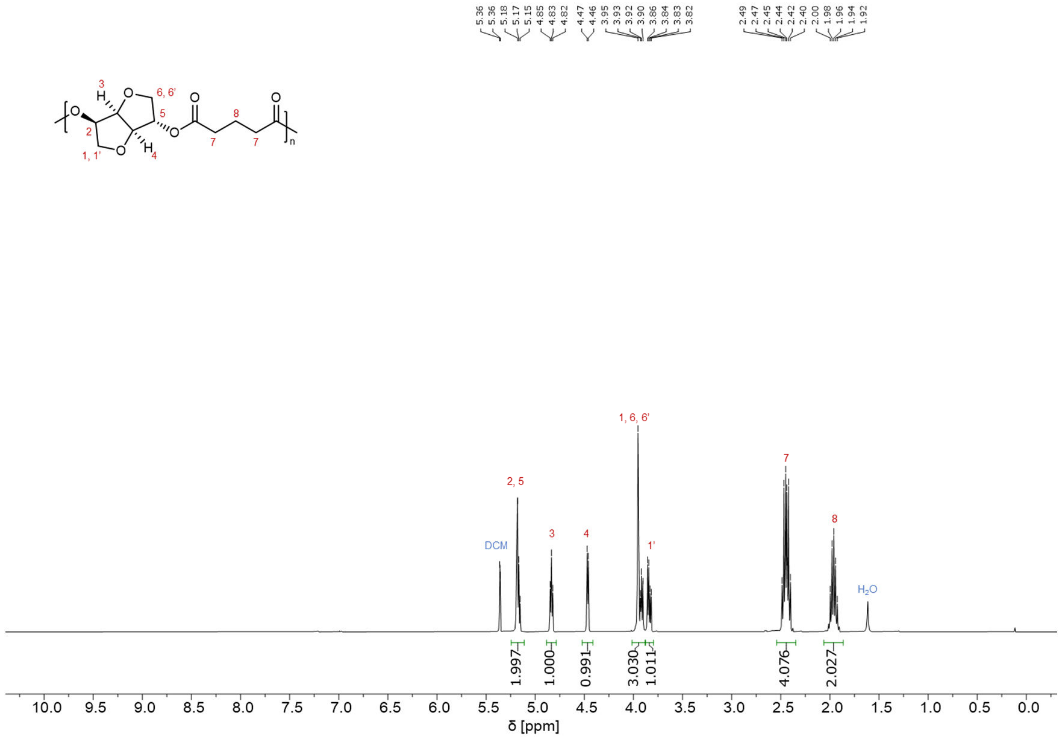

Supplementary Figure 11. ^1H NMR spectrum (298 K, 500 MHz) of poly(isosorbide glutarate) in DCM-d2.

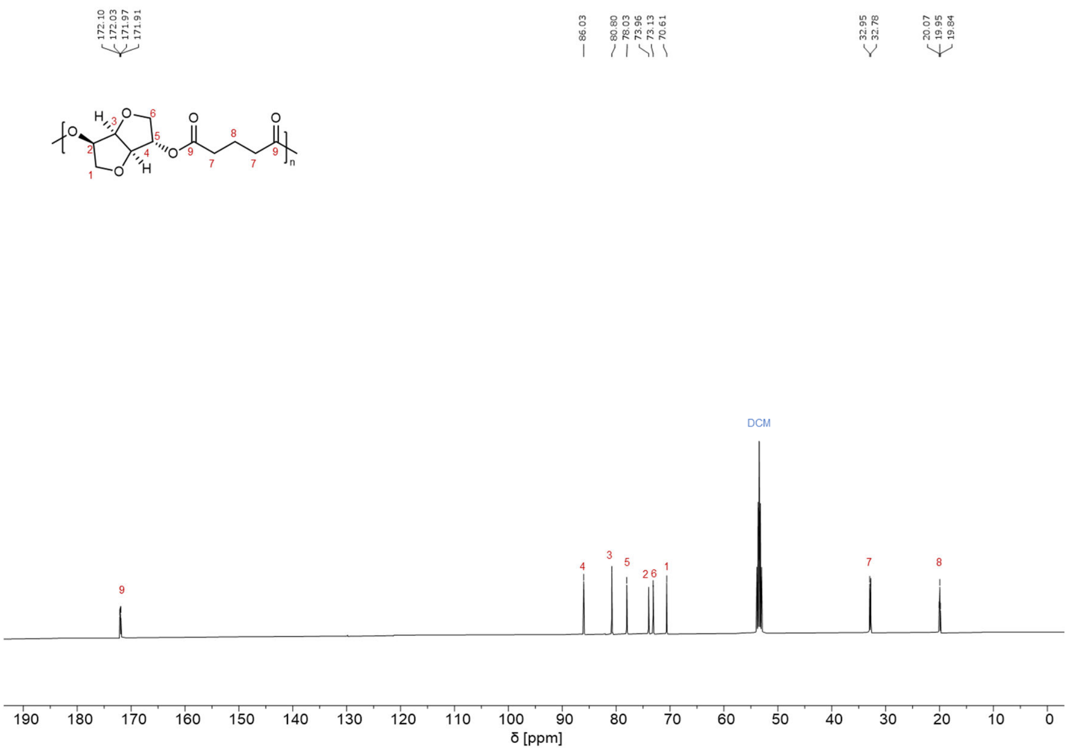

Supplementary Figure 12. ^{13}C NMR spectrum (298 K, 125 MHz) of poly(isosorbide glutarate) in DCM-d2. The spectrum is comparable to the spectrum reported for the same compound in $CDCl_3$ by Okada *et al.*[2].

Additional characterization of poly(isosorbide adipate) (PIsAd)

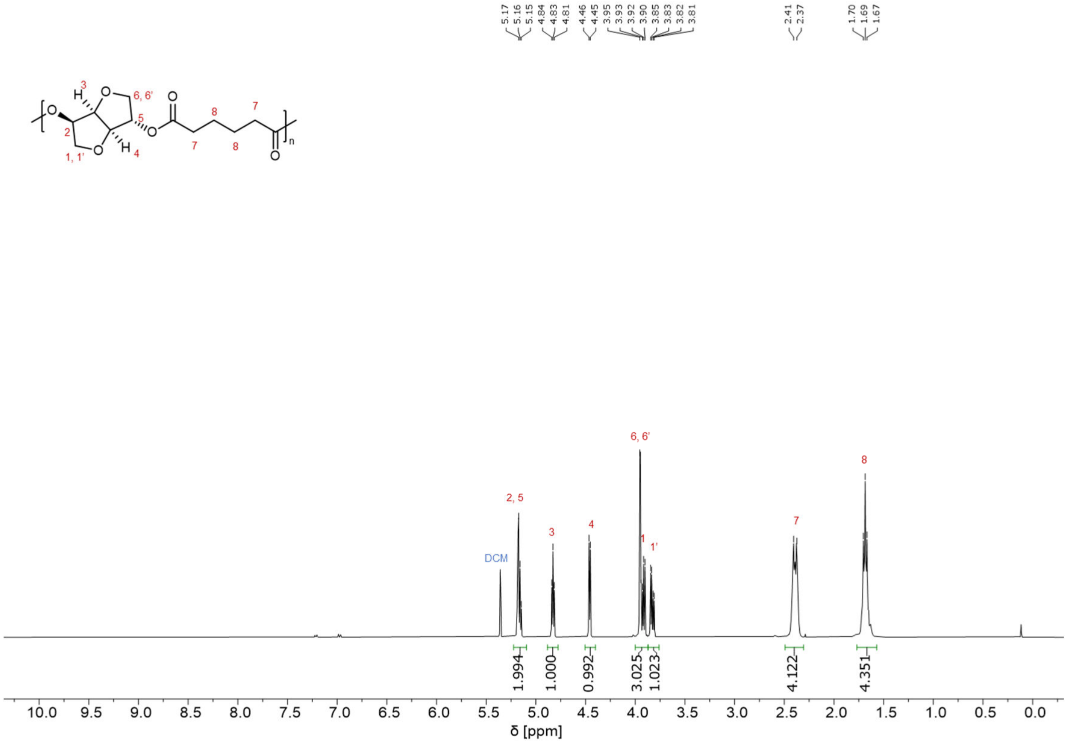

Supplementary Figure 13. ^{1}H NMR spectrum (298 K, 500 MHz) of poly(isosorbide adipate) in DCM-d2.

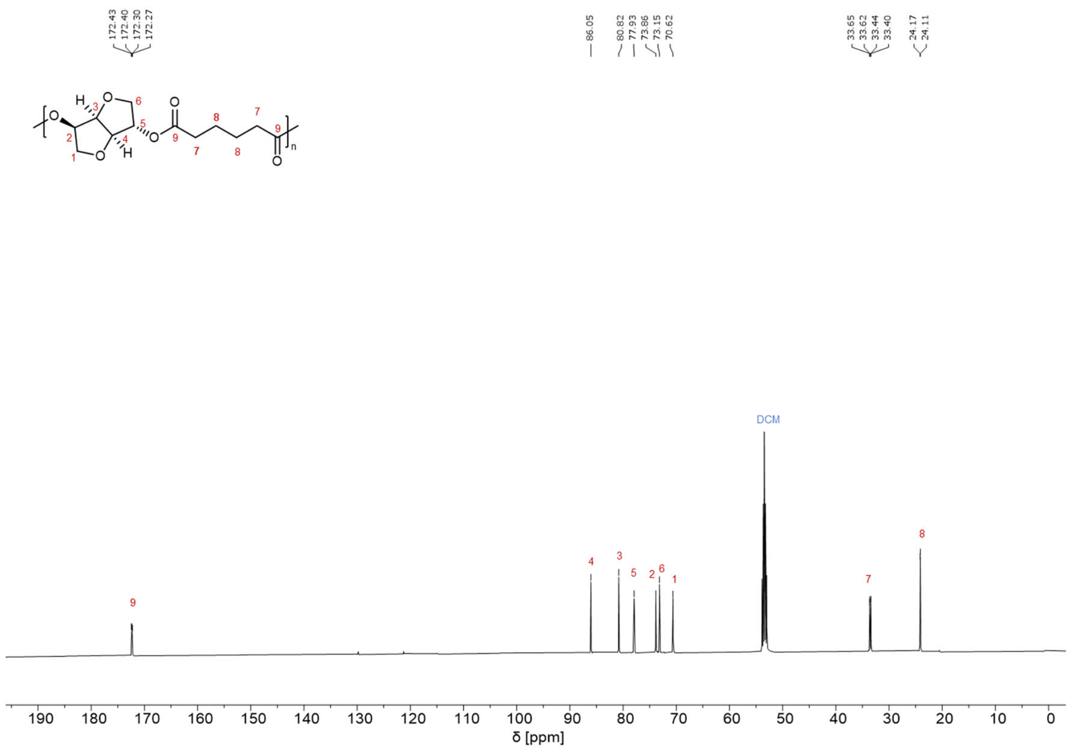

Supplementary Figure 14. ^{13}C NMR spectrum (298 K, 125 MHz) of poly(isosorbide adipate) in DCM-d2.

Additional characterization of poly(isosorbide-1,4-cyclohexanedicarboxylate) (PIsCyc)

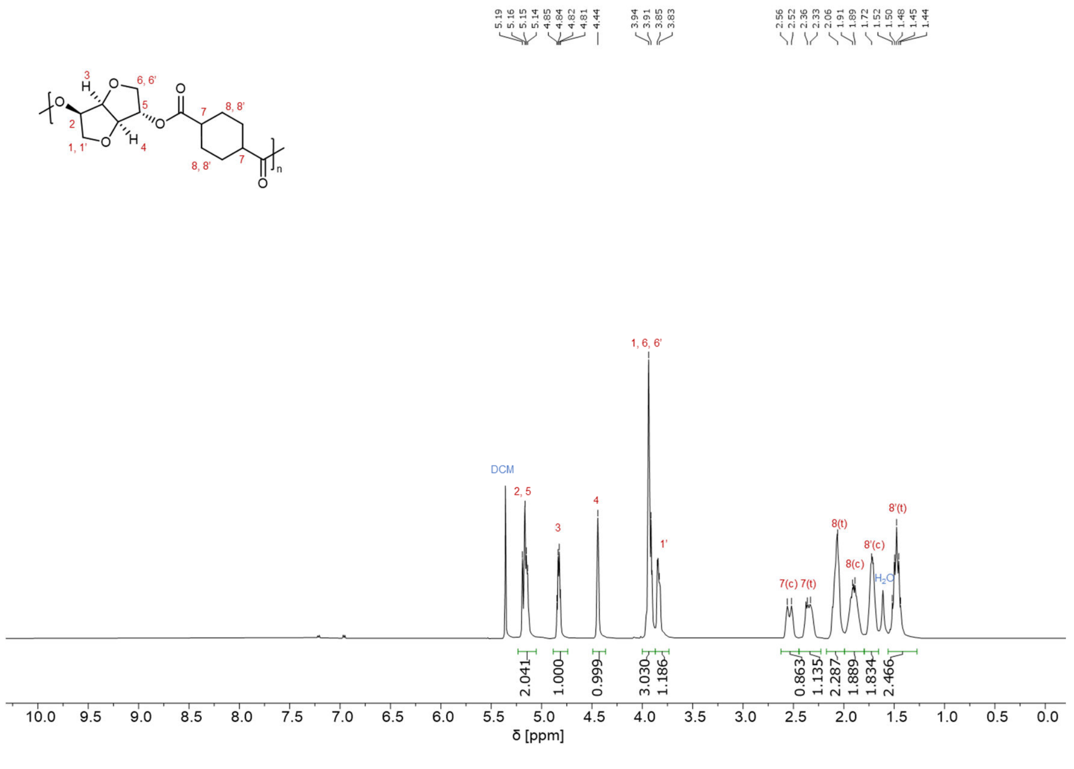

Supplementary Figure 15. ^{1}H NMR spectrum (298 K, 500 MHz) of poly(isosorbide-1,4-cyclohexanedicarboxylate) in DCM-d2.

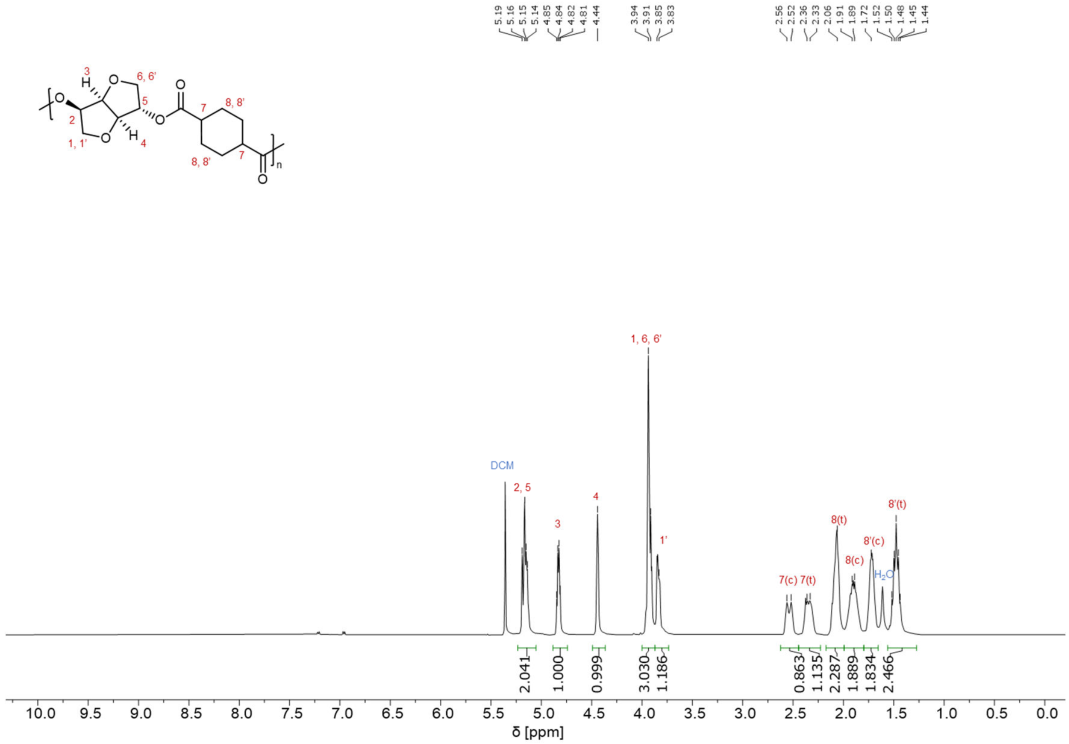

Supplementary Figure 16. ^{13}C NMR spectrum (298 K, 125 MHz) of poly(isosorbide-1,4-cyclohexanedicarboxylate) in DCM-d2.

Poly(isosorbide-1,4-cyclohexanedicarboxylate) has been characterized by 2D NMR spectroscopy by Yoon *et al.*[3]. 2D NMR spectra recorded in DCM-d2 were comparable to those reported by Yoon *et al.* except for the absence of signals corresponding to 1,4-sorbitan (not detected in our samples).

Additional characterization of poly(isosorbide diglycolate) (PIsDga)

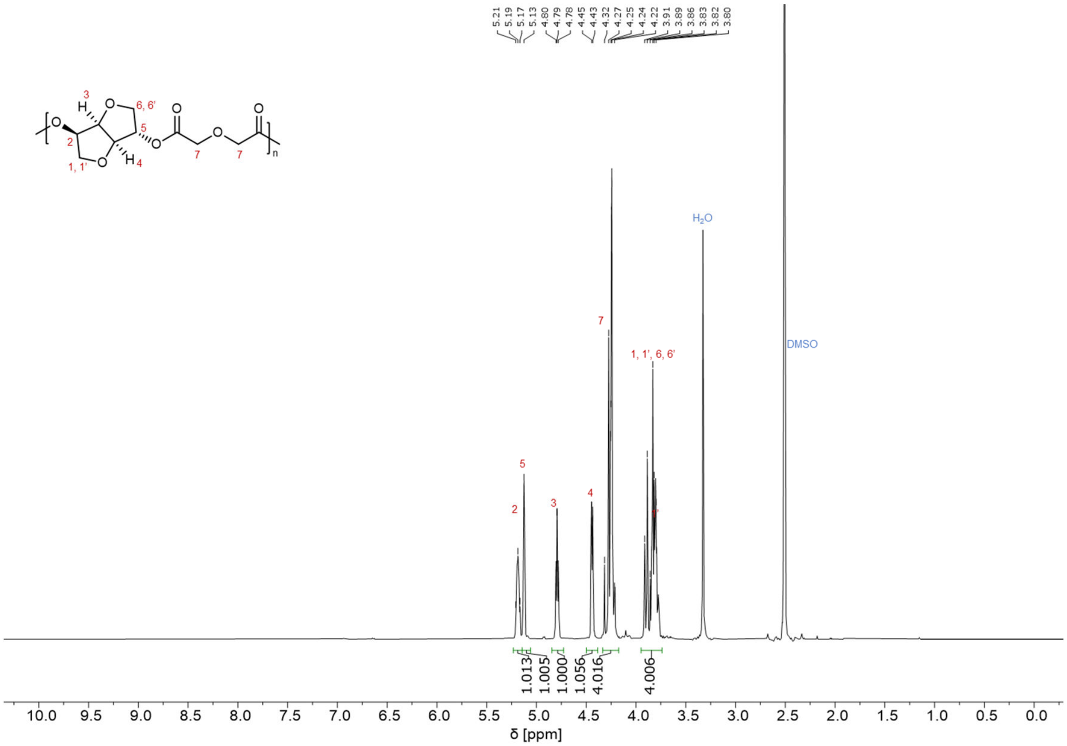

Supplementary Figure 17. ^{1}H NMR spectrum (298 K, 500 MHz) of poly(isosorbide diglycolate) in DMSO-d6.

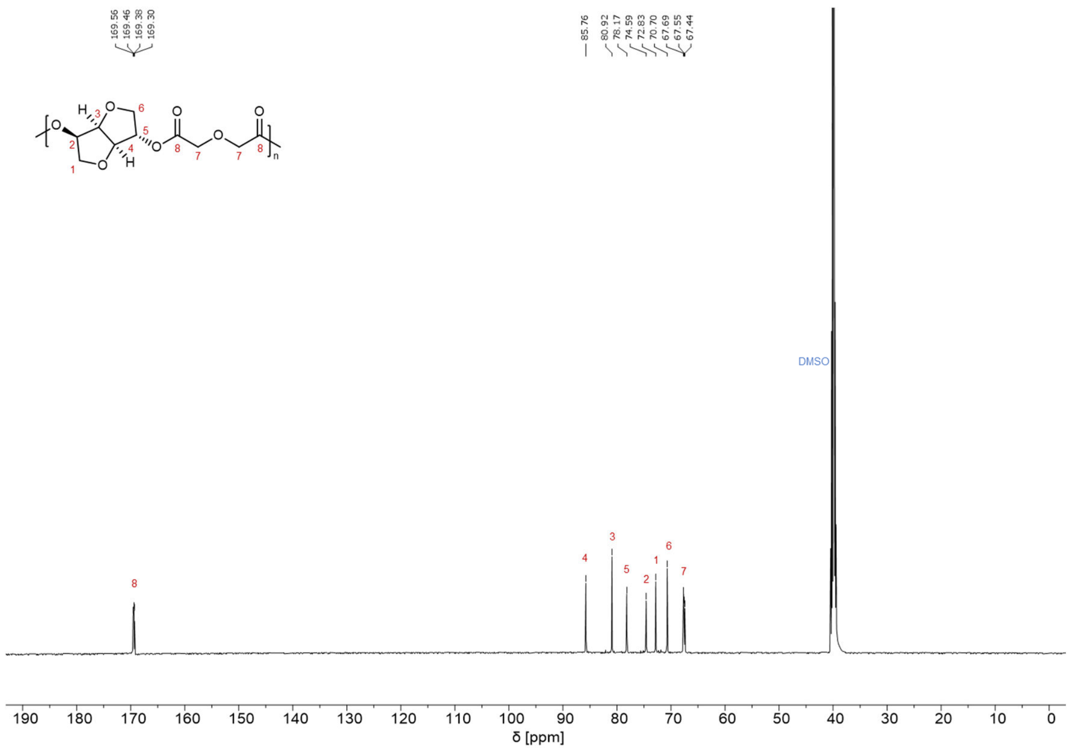

Supplementary Figure 18. ^{13}C NMR spectrum (298 K, 125 MHz) of poly(isosorbide diglycolate) in DMSO-d6.

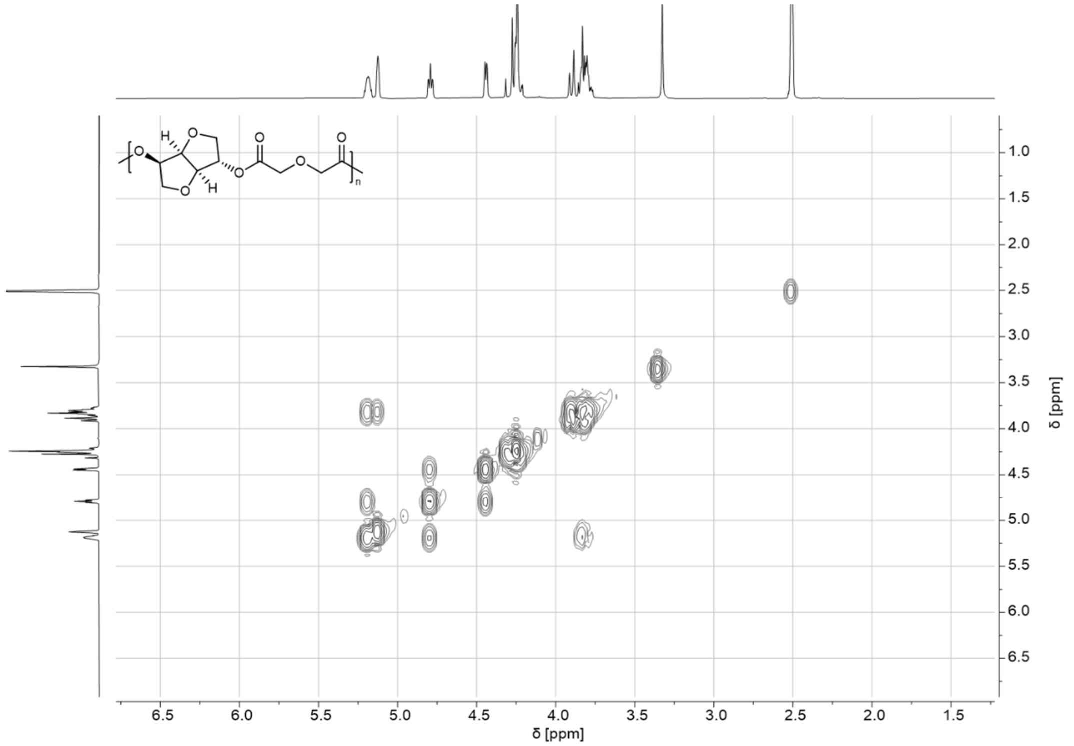

Supplementary Figure 19. ^{1}H-^{1}H COSY NMR spectrum (298 K, 500 MHz) of poly(isosorbide diglycolate) in DMSO-d6.

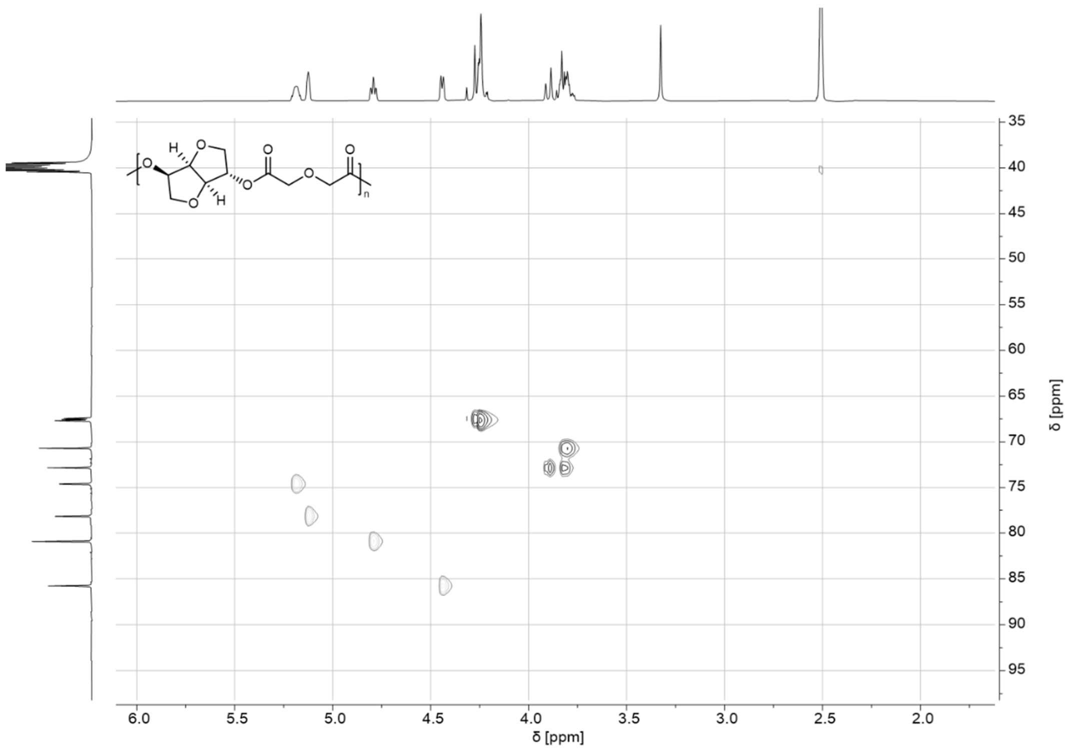

Supplementary Figure 20. ^{1}H-^{13}C HSQC NMR spectrum (298 K, 500/125 MHz) of poly(isosorbide diglycolate) in DMSO-d6.

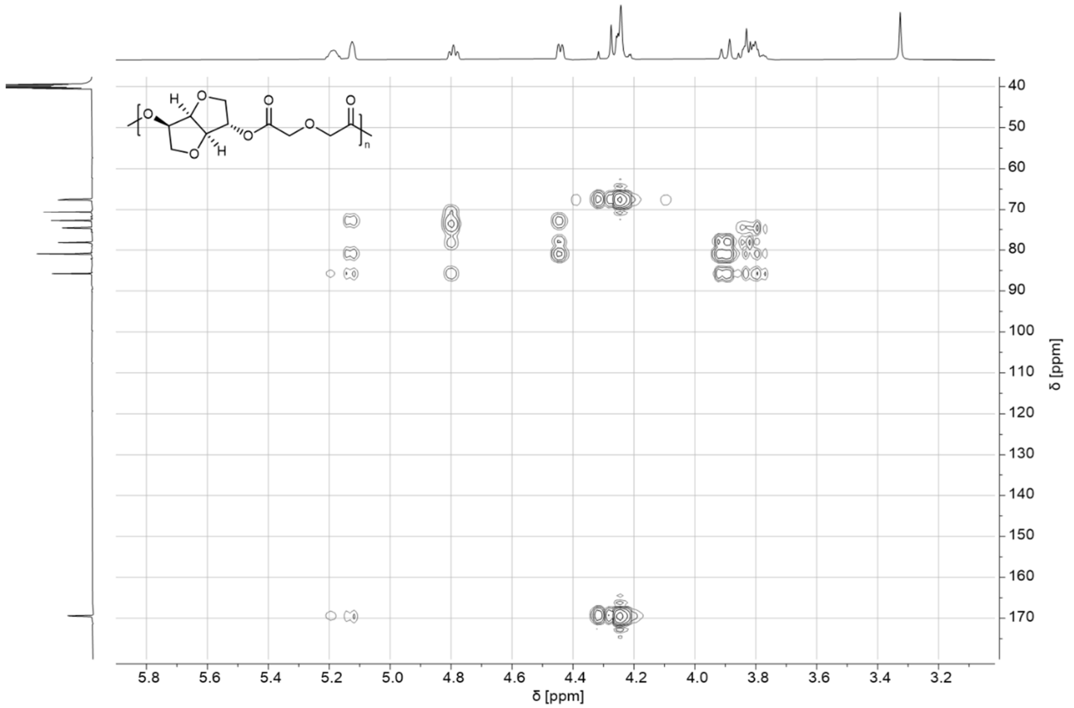

Supplementary Figure 21. ^{1}H-^{13}C HMBC NMR spectrum (298 K, 500/125 MHz) of poly(isosorbide diglycolate) in DMSO-d6.

Additional characterization of poly(isosorbide thiodiglycolate) (PIsThd)

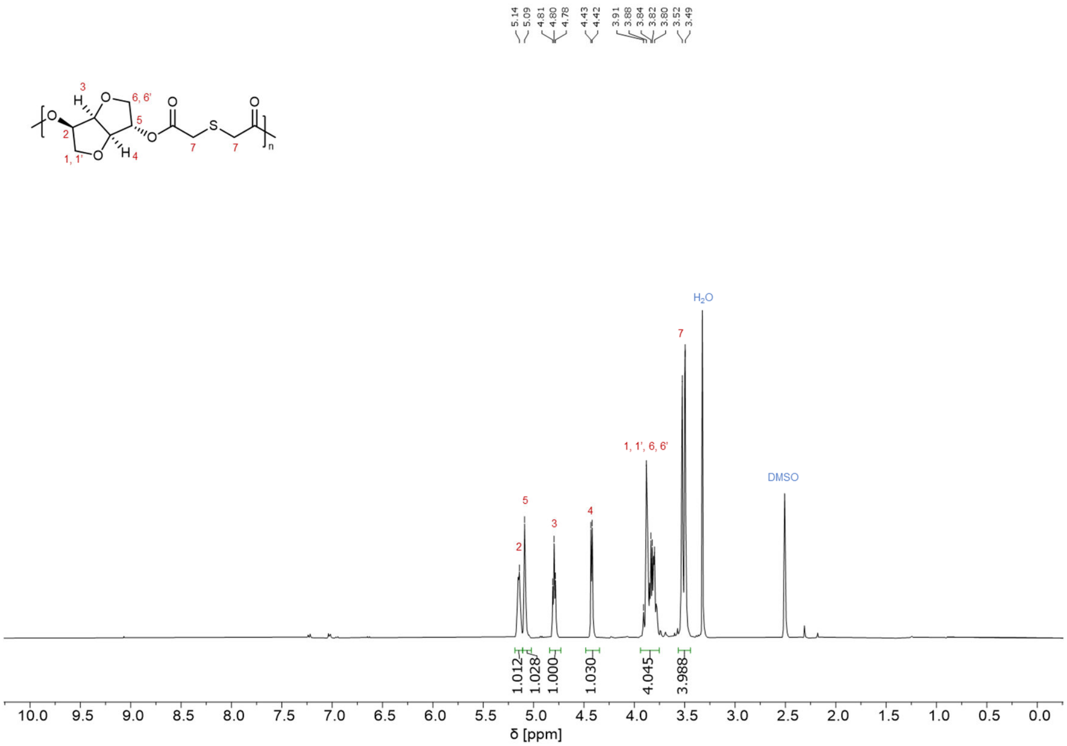

Supplementary Figure 22. ^{1}H NMR spectrum (298 K, 500 MHz) of poly(isosorbide thiodiglycolate) in DMSO-d6.

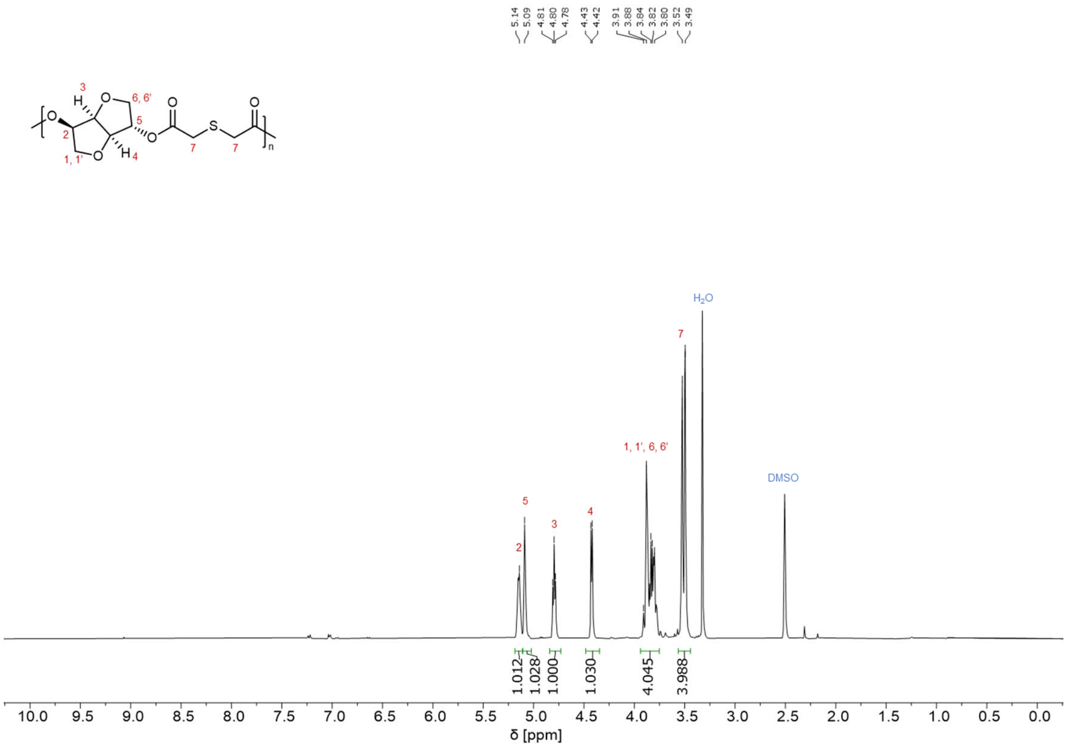

Supplementary Figure 23. ^{13}C NMR spectrum (298 K, 125 MHz) of poly(isosorbide thiodiglycolate) in DMSO-d6.

Additional characterization of poly(isomannide succinate) (PImSu)

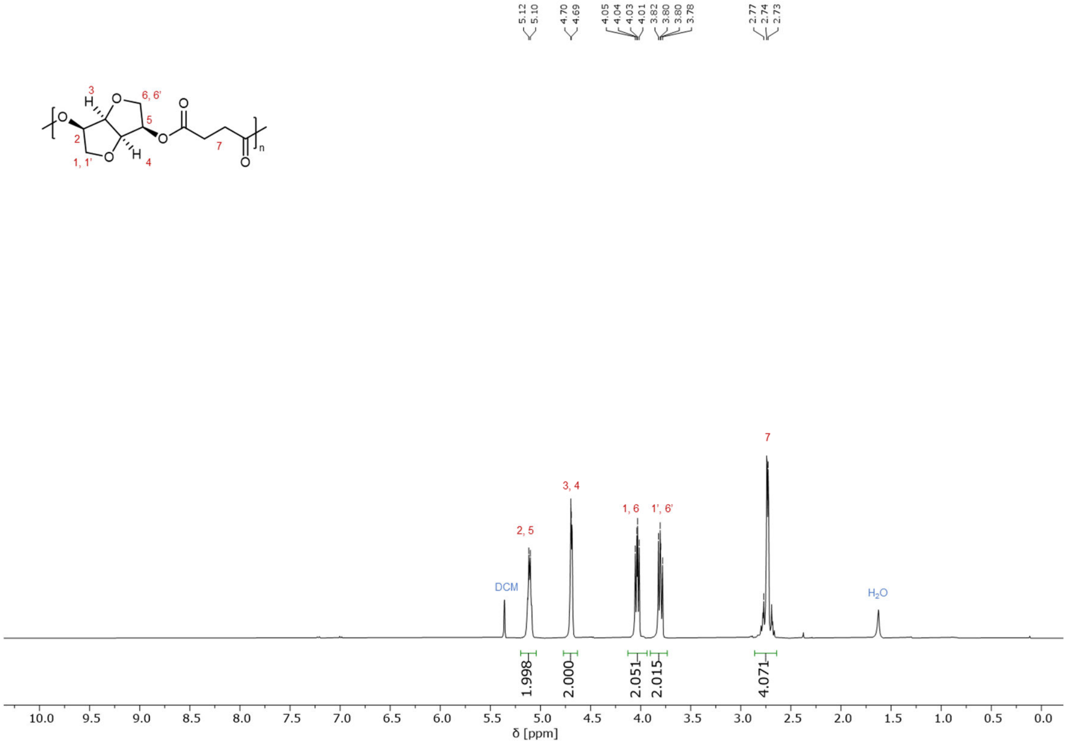

Supplementary Figure 24. ^{1}H NMR spectrum (298 K, 500 MHz) of poly(isomannide succinate) in DCM-d2.

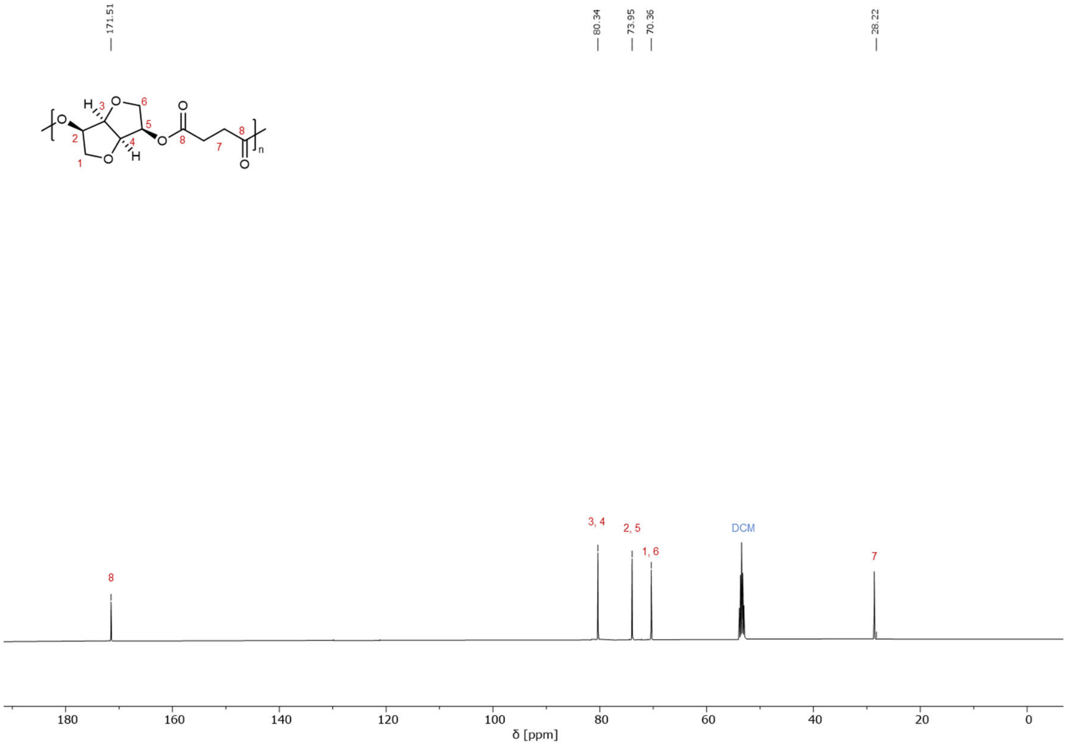

Supplementary Figure 25. ^{13}C NMR spectrum (298 K, 125 MHz) of poly(isomannide succinate) in DCM-d2.

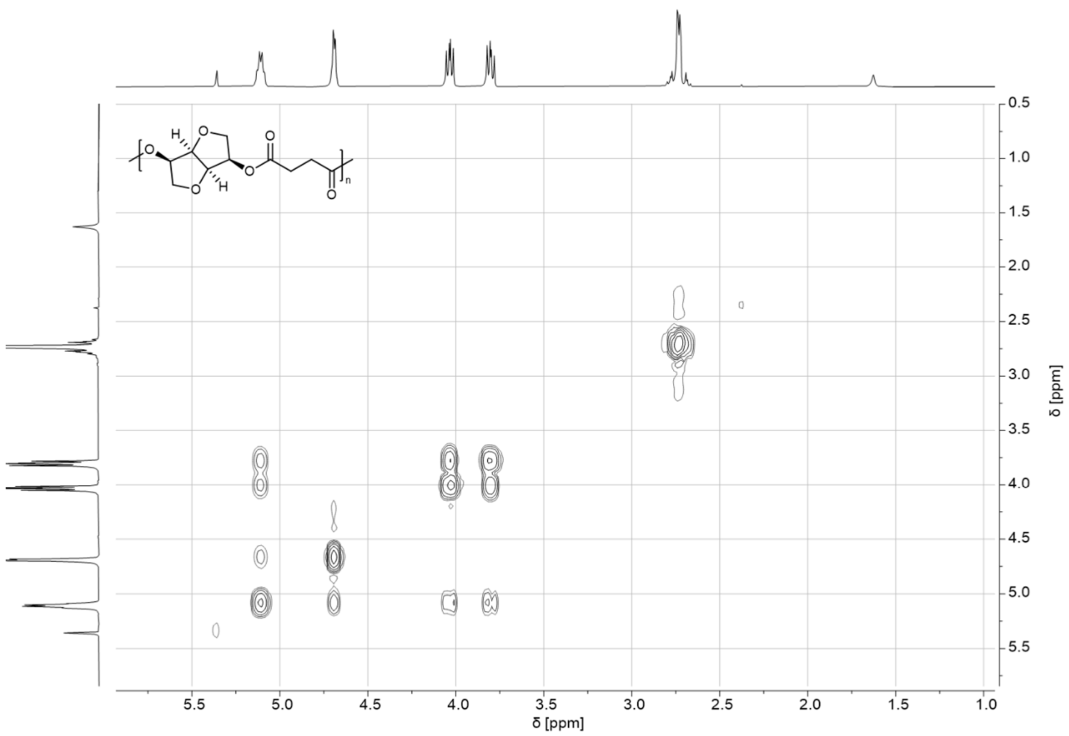

Supplementary Figure 26. ^{1}H-^{1}H COSY NMR spectrum (298 K, 500 MHz) of poly(isomannide succinate) in DCM-d2.

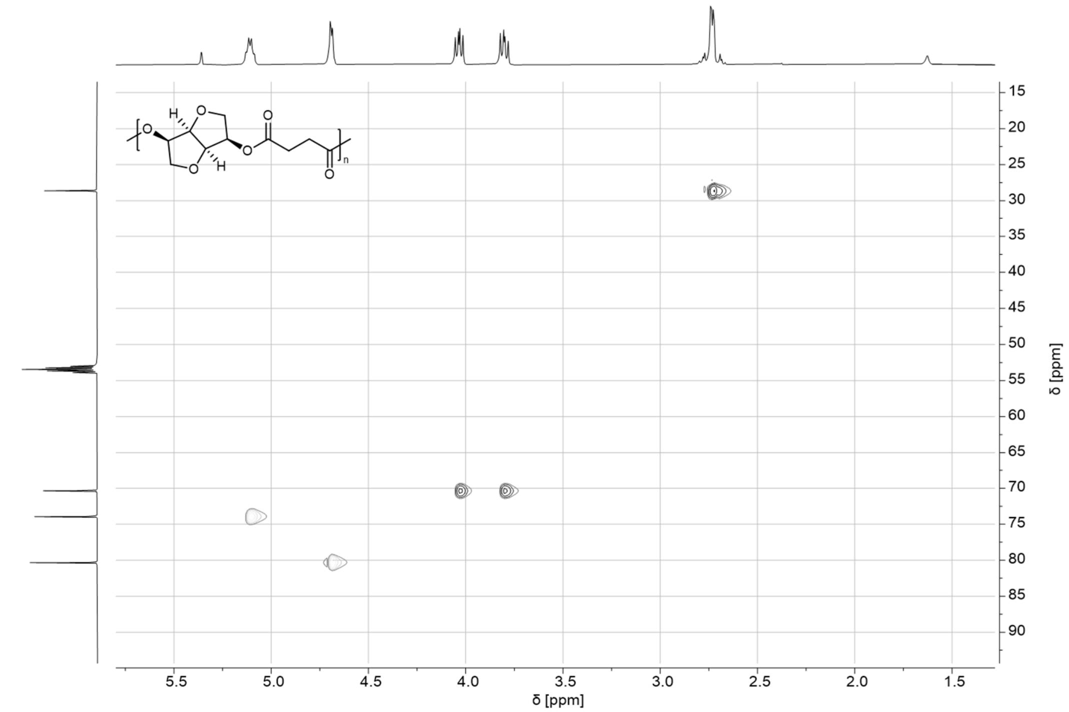

Supplementary Figure 27. ^{1}H-^{13}C HSQC NMR spectrum (298 K, 500/125 MHz) of poly(isomannide succinate) in DCM-d2.

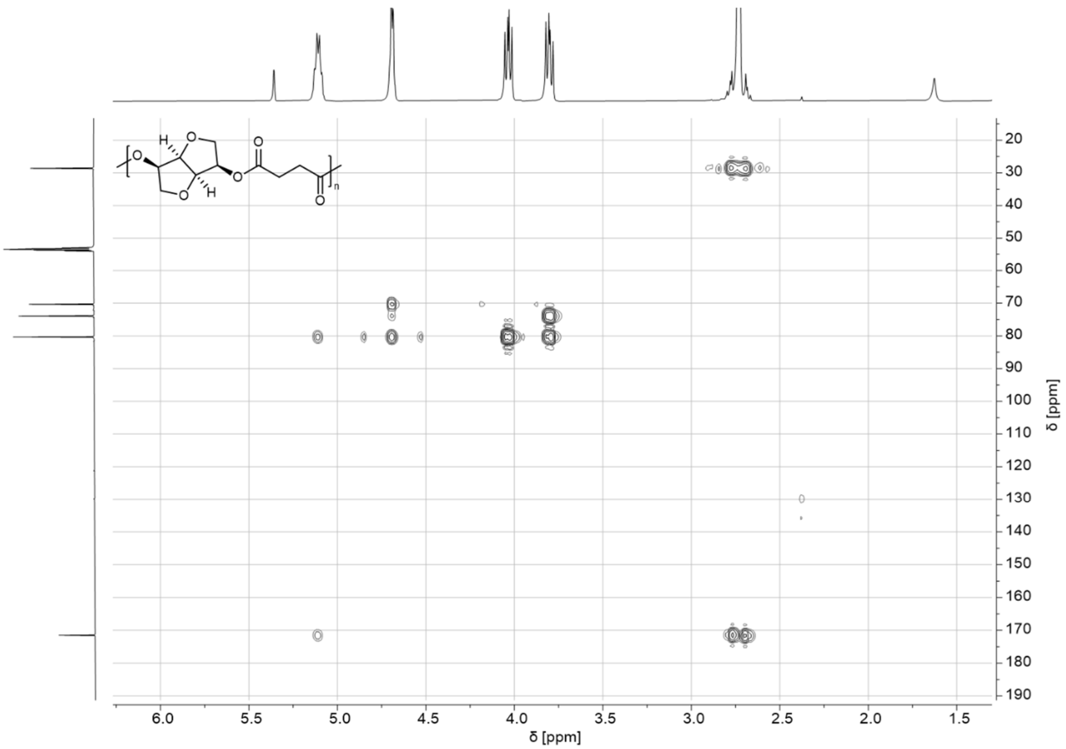

Supplementary Figure 28. ^{1}H-^{13}C HMBC NMR spectrum (298 K, 500/125 MHz) of poly(isomannide succinate) in DCM-d2.

Additional characterization of poly(isomannide glutarate) (PImGlu)

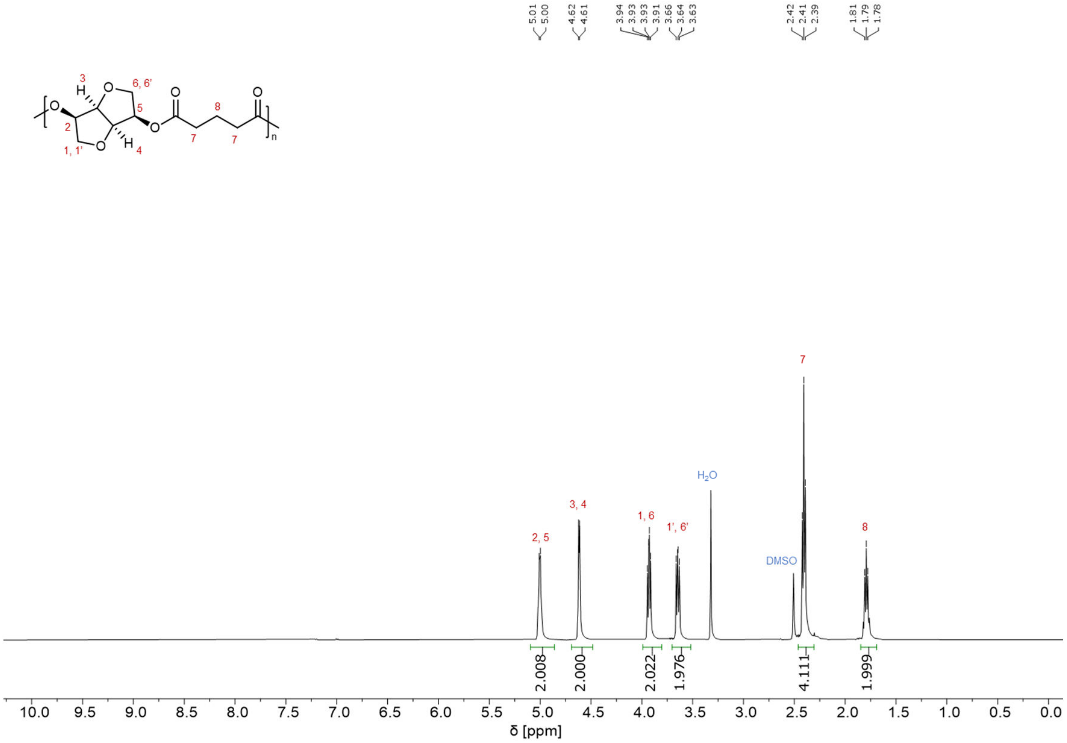

Supplementary Figure 29. ^{1}H NMR spectrum (298 K, 500 MHz) of poly(isomannide glutarate) in DMSO-d6.

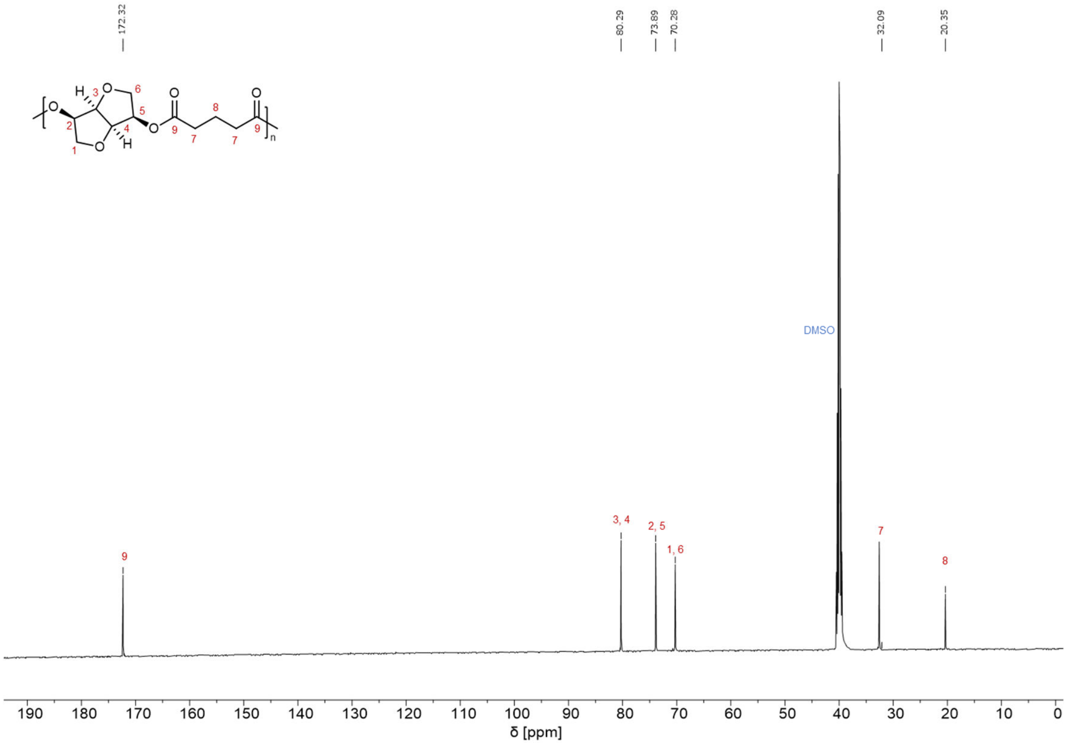

Supplementary Figure 30. ^{13}C NMR spectrum (298 K, 125 MHz) of poly(isomannide glutarate) in DMSO-d6.

Additional characterization of poly(isomannide adipate) (PImAd)

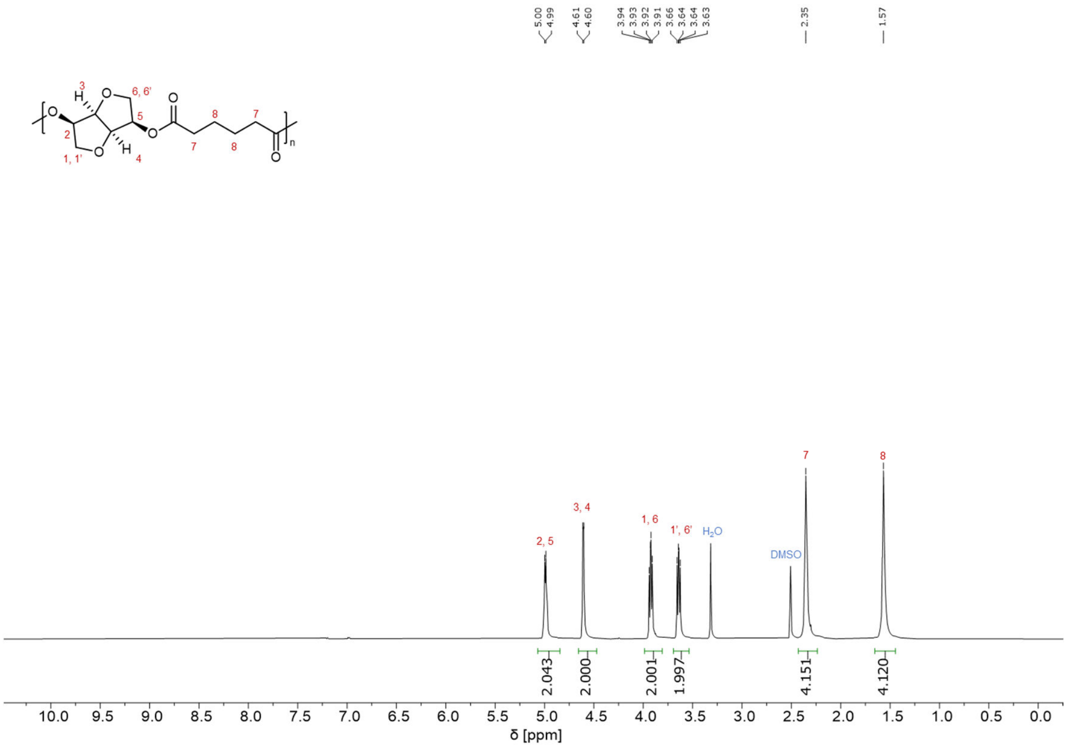

Supplementary Figure 31. ^{1}H NMR spectrum (298 K, 500 MHz) of poly(isomannide adipate) in DMSO-d6.

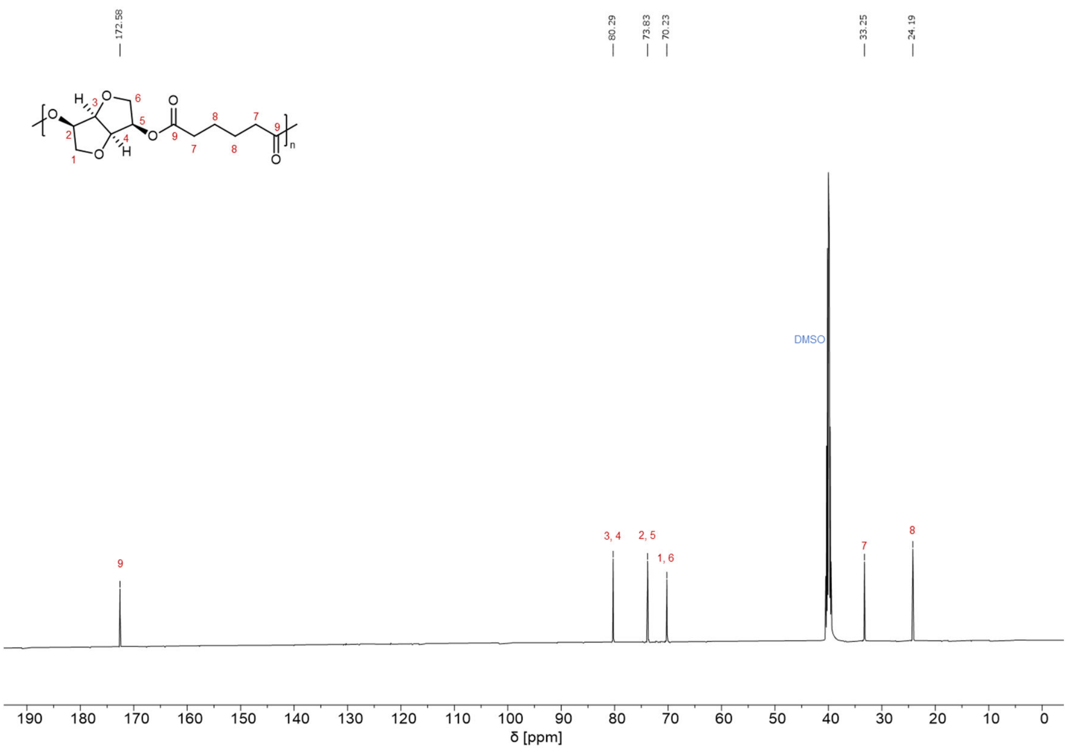

Supplementary Figure 32. ^{13}C NMR spectrum (298 K, 125 MHz) of poly(isomannide adipate) in DMSO-d6.

Additional characterization of poly(isomannide-1,4-cyclohexanedicarboxylate) (PImCyc)

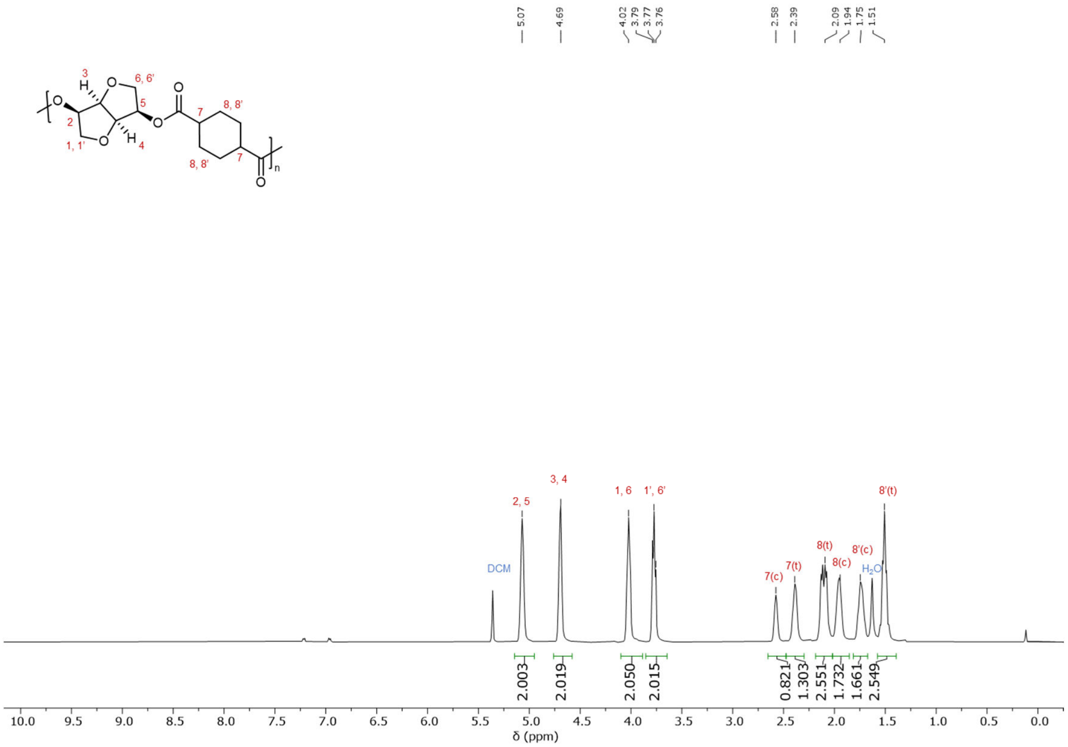

Supplementary Figure 33 ^{1}H NMR spectrum (298 K, 500 MHz) of poly(isomannide-1,4-cyclohexanedicarboxylate) in DCM-d2.

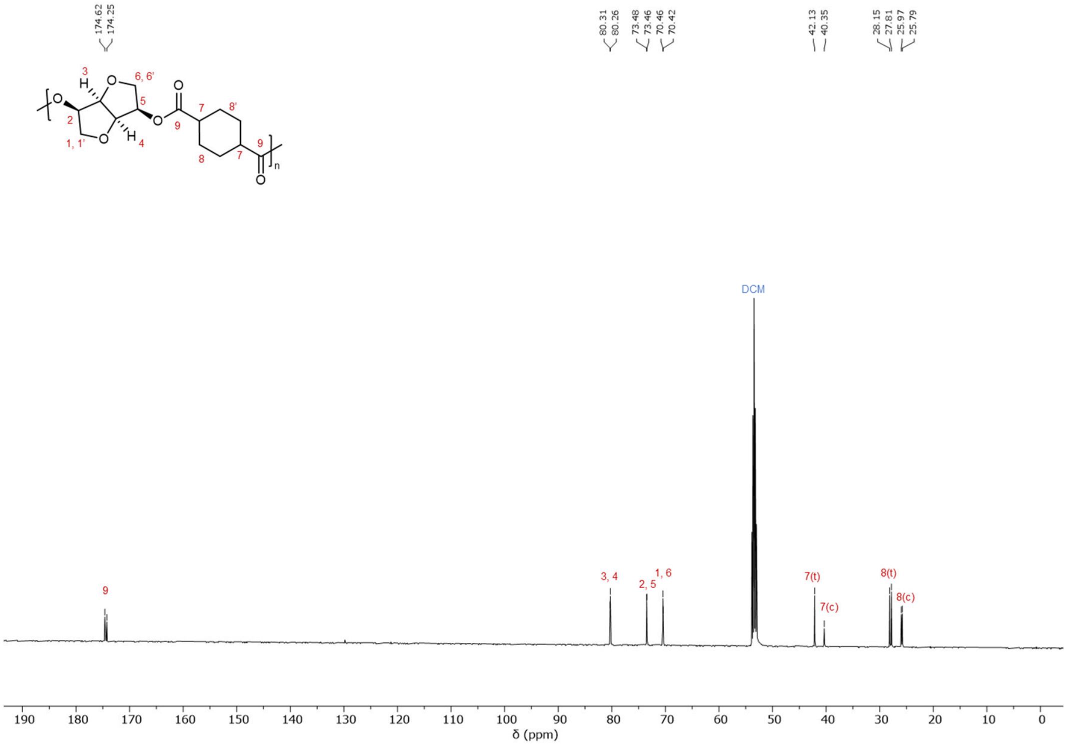

Supplementary Figure 34. ^{13}C NMR spectrum (298 K, 125 MHz) of poly(isomannide-1,4-cyclohexanedicarboxylate) in DCM-d2.

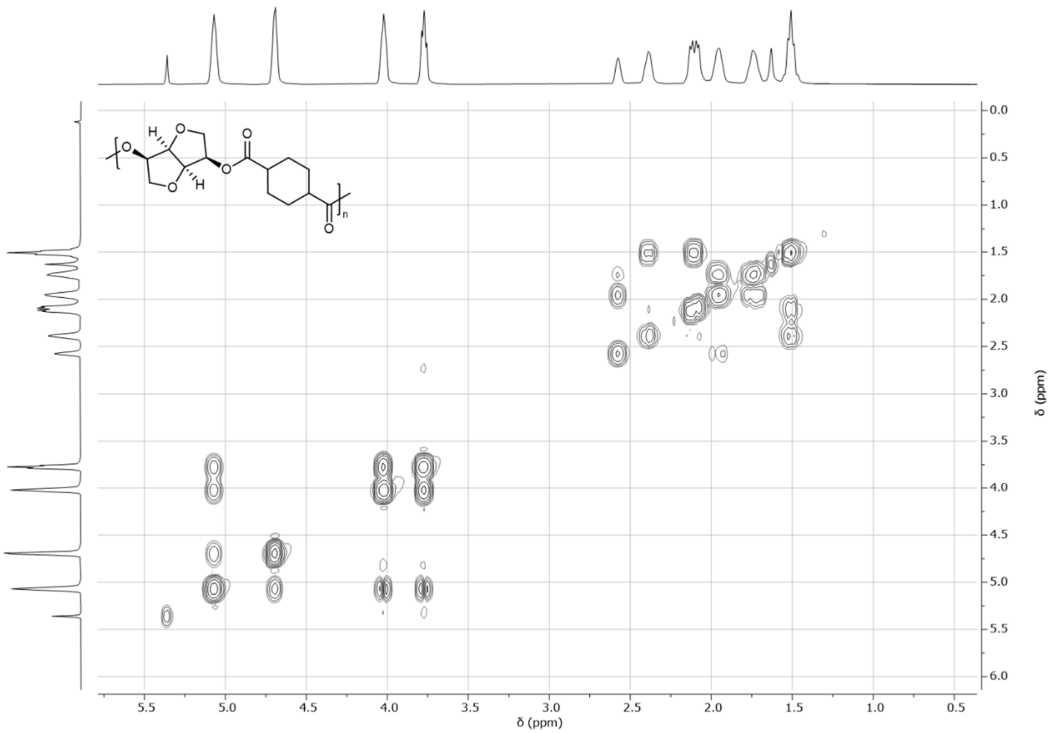

Supplementary Figure 35. ^{1}H-^{1}H COSY NMR spectrum (298 K, 500 MHz) of poly(isomannide-1,4-cyclohexanedicarboxylate) in DCM-d2.

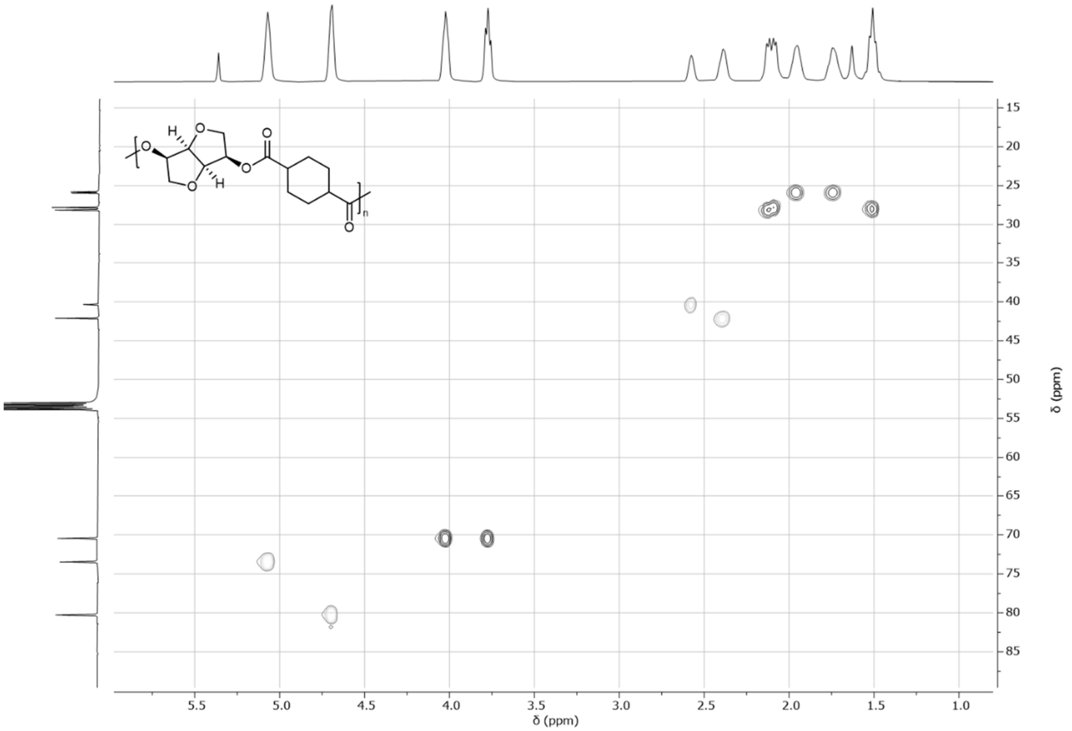

Supplementary Figure 36. ^{1}H-^{13}C HSQC NMR spectrum (298 K, 500/125 MHz) of poly(isomannide-1,4-cyclohexanedicarboxylate) in DCM-d2.

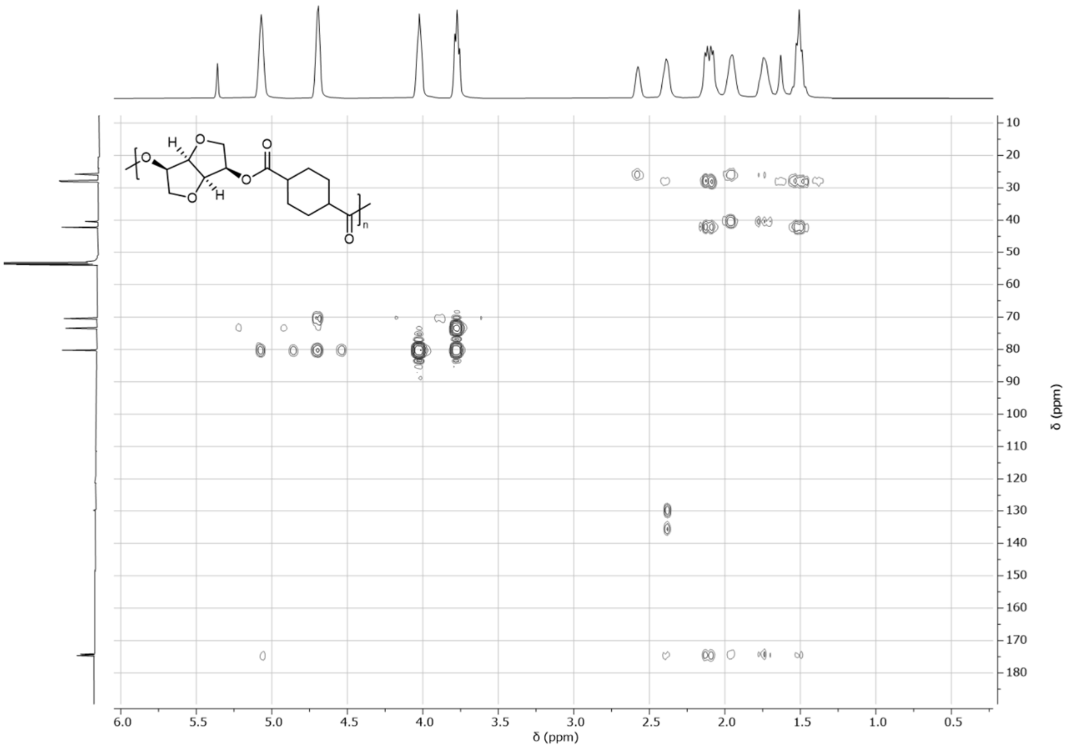

Supplementary Figure 37. ^{1}H-^{13}C HMBC NMR spectrum (298 K, 500/125 MHz) of poly(isomannide-1,4-cyclohexanedicarboxylate) in DCM-d2.

Additional characterization of poly(isomannide diglycolate) (PImDga)

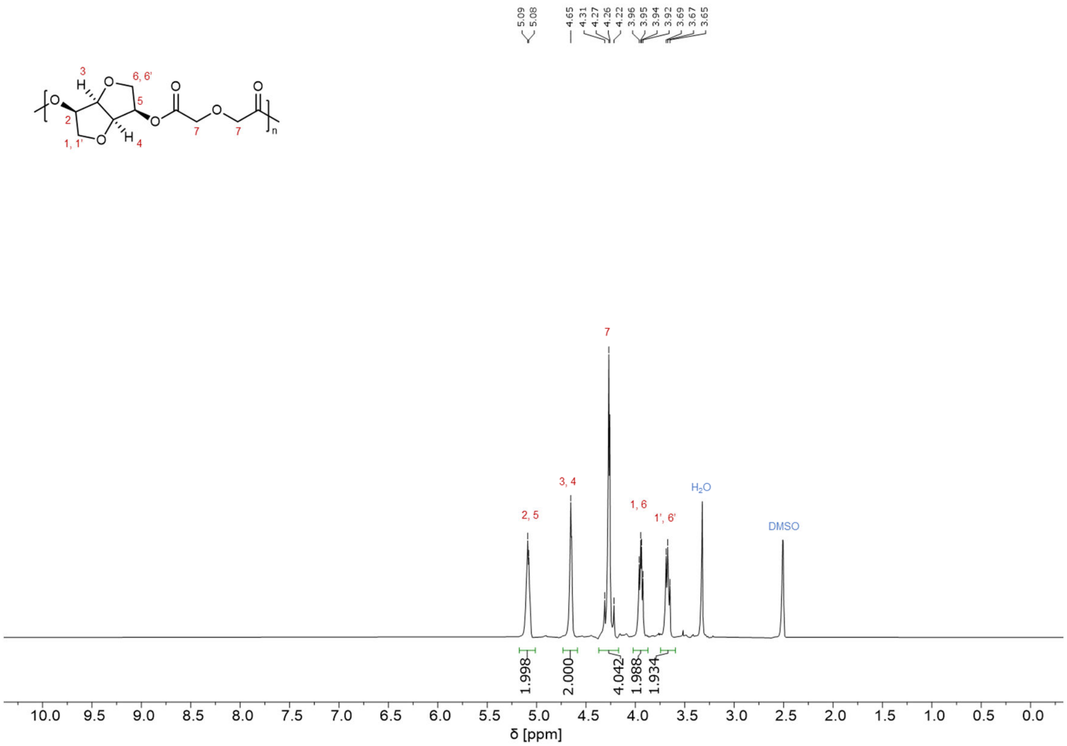

Supplementary Figure 38. ^{1}H NMR spectrum (298 K, 500 MHz) of poly(isomannide diglycolate) in DMSO-d6.

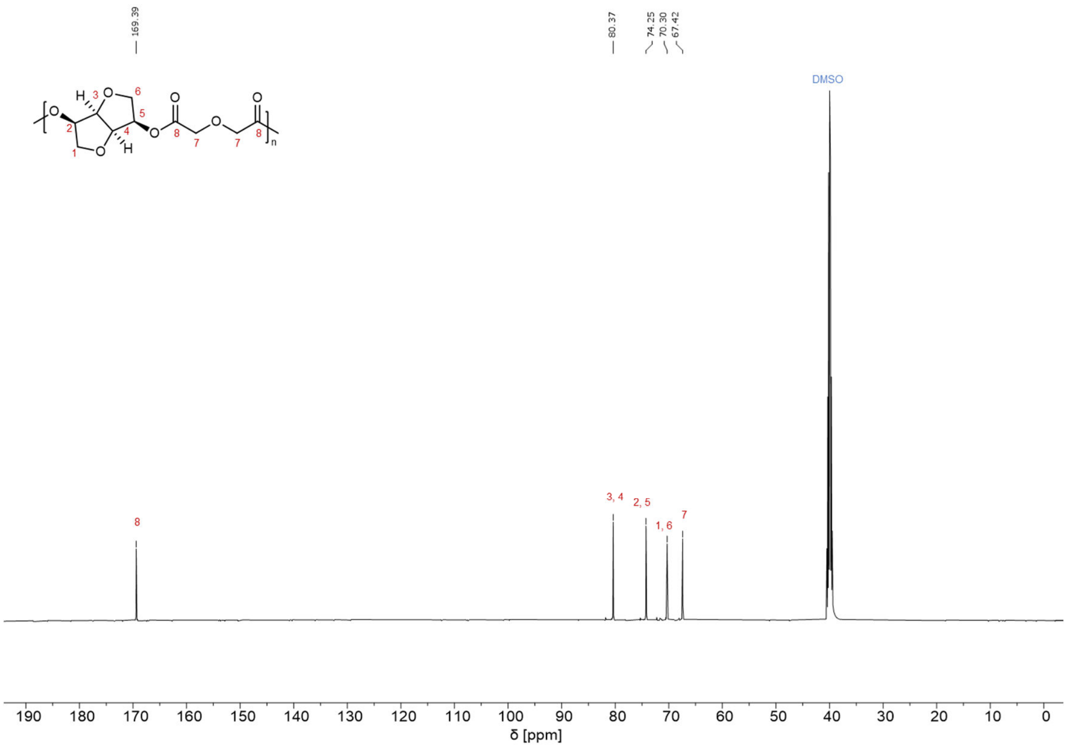

Supplementary Figure 39. ^{13}C NMR spectrum (298 K, 125 MHz) of poly(isomannide diglycolate) in DMSO-d6.

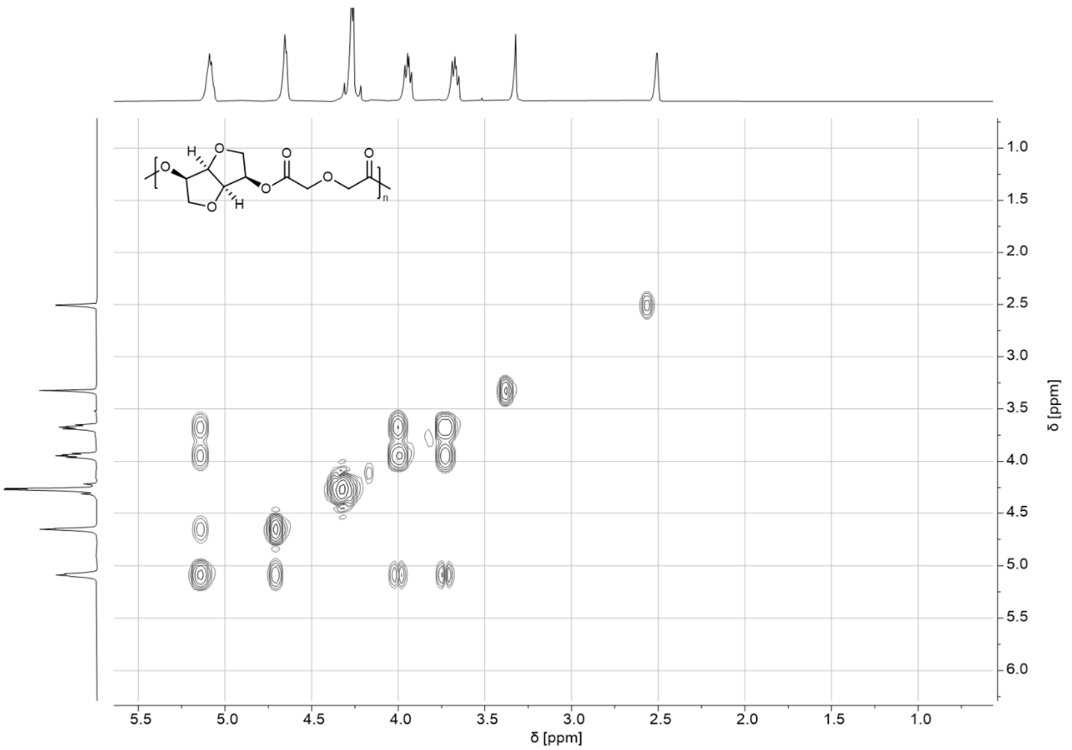

Supplementary Figure 40. ^{1}H-^{1}H COSY NMR spectrum (298 K, 500 MHz) of poly(isomannide diglycolate) in DMSO-d6.

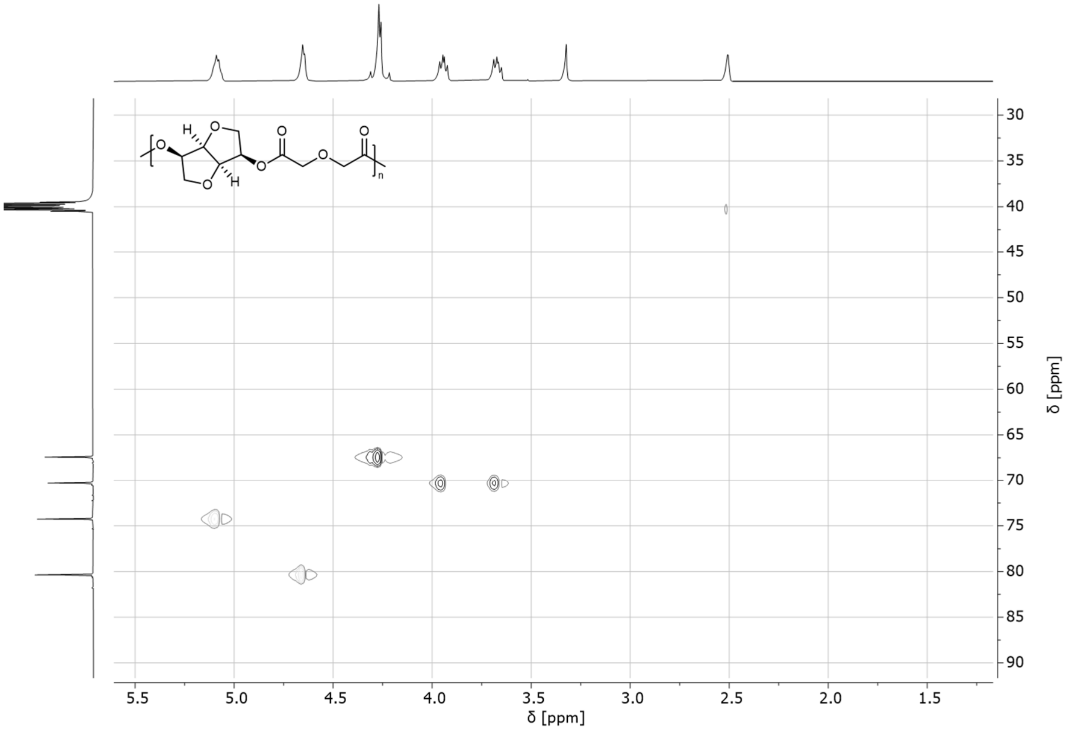

Supplementary Figure 41. ^{1}H-^{13}C HSQC NMR spectrum (298 K, 500/125 MHz) of poly(isomannide diglycolate) in DMSO-d6.

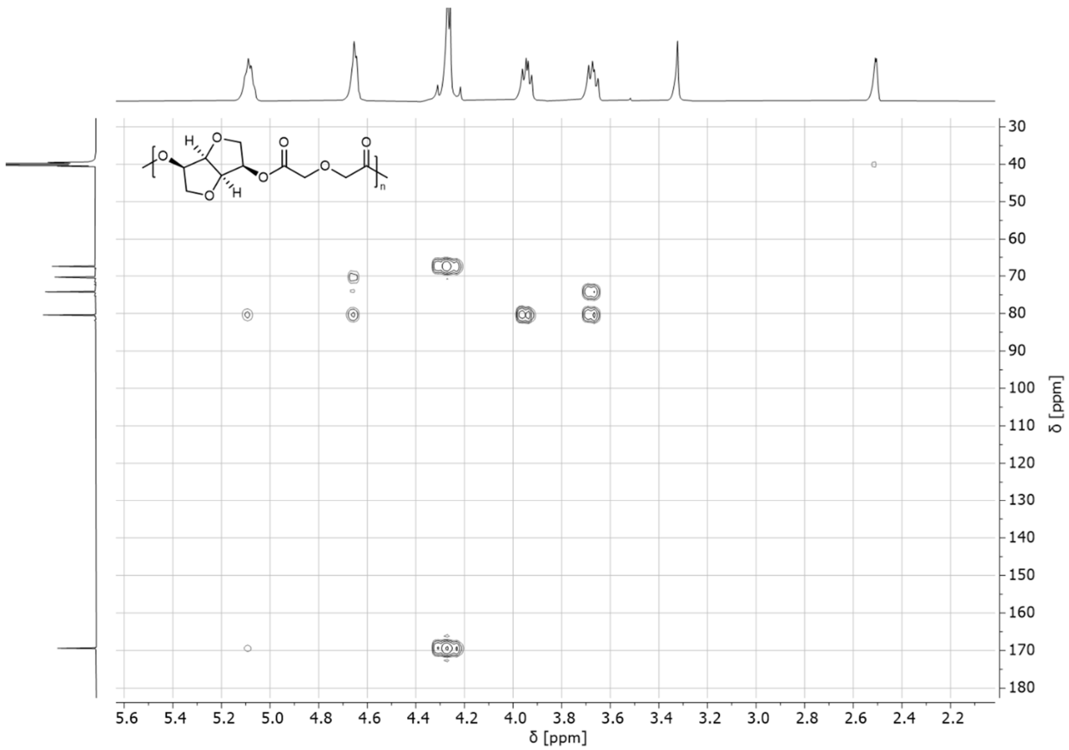

Supplementary Figure 42. ^{1}H-^{13}C HMBC NMR spectrum (298 K, 500/125 MHz) of poly(isomannide diglycolate) in DMSO-d6.

DSC traces of isosorbide-based polyesters

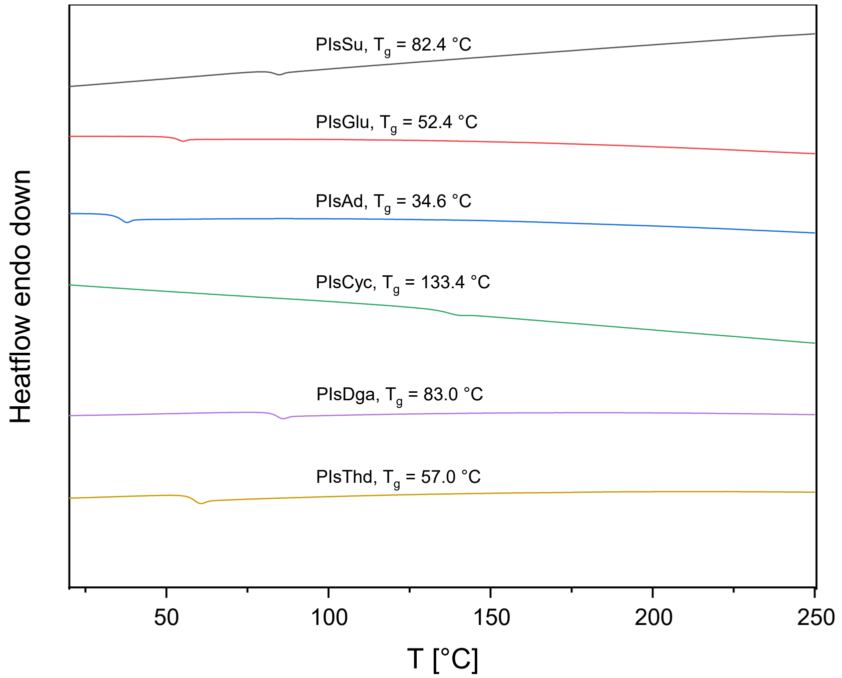

Supplementary Figure 43. DSC traces of isosorbide-based polyesters recorded during the second heating cycle.

DSC traces of isomannide-based polyesters

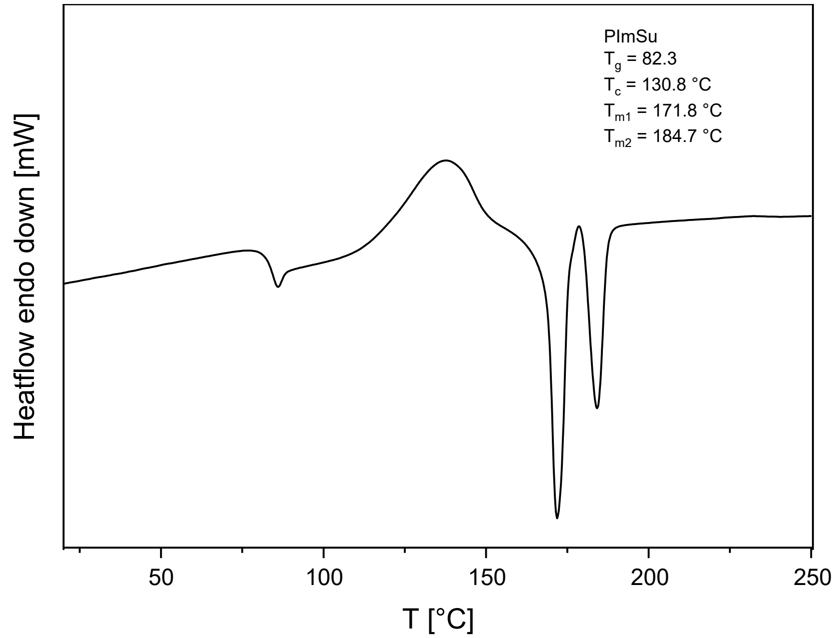

Supplementary Figure 44. DSC trace of PImSu recorded during the second heating cycle.

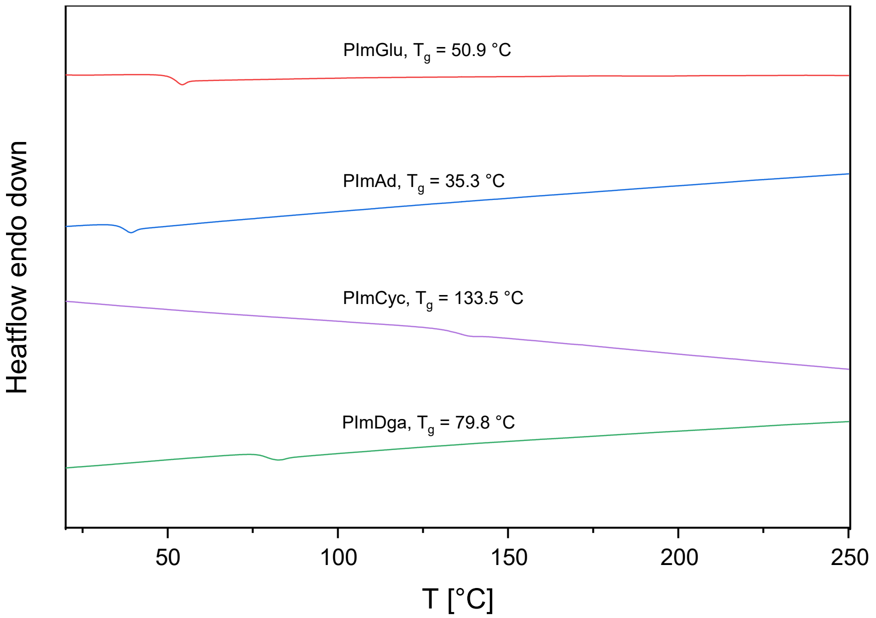

Supplementary Figure 45. DSC traces of PImGlu, PImAd, PImCyc and PImDga recorded during the second heating cycle.

Thermal stability of isosorbide- and isomannide-based polyesters

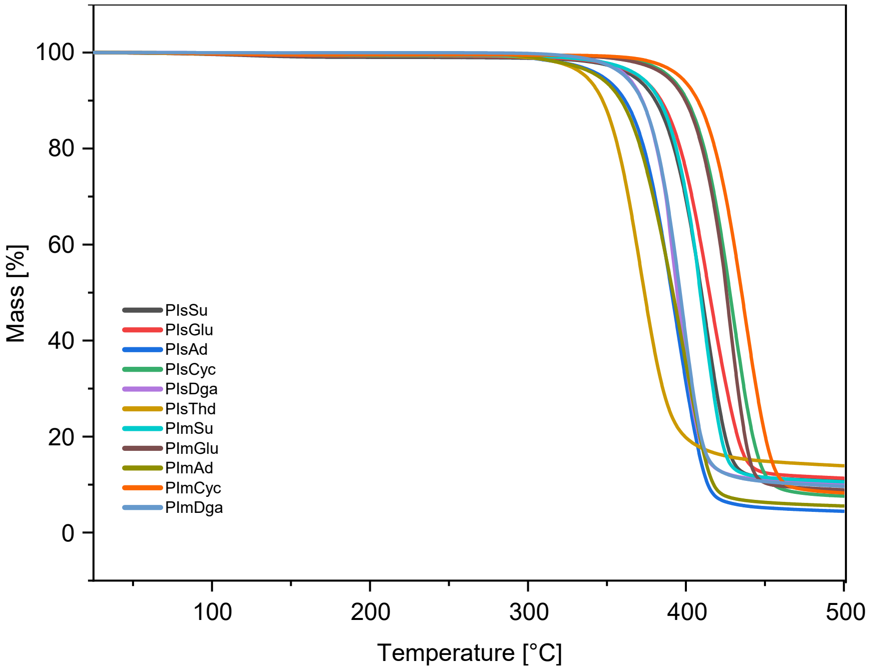

Supplementary Figure 46. TGA traces of all synthesized polyesters. For $T_{5\%d}$ values, see Supplementary Table 3.

O_2 and H_2O permeability curves for isosorbide-based polyesters

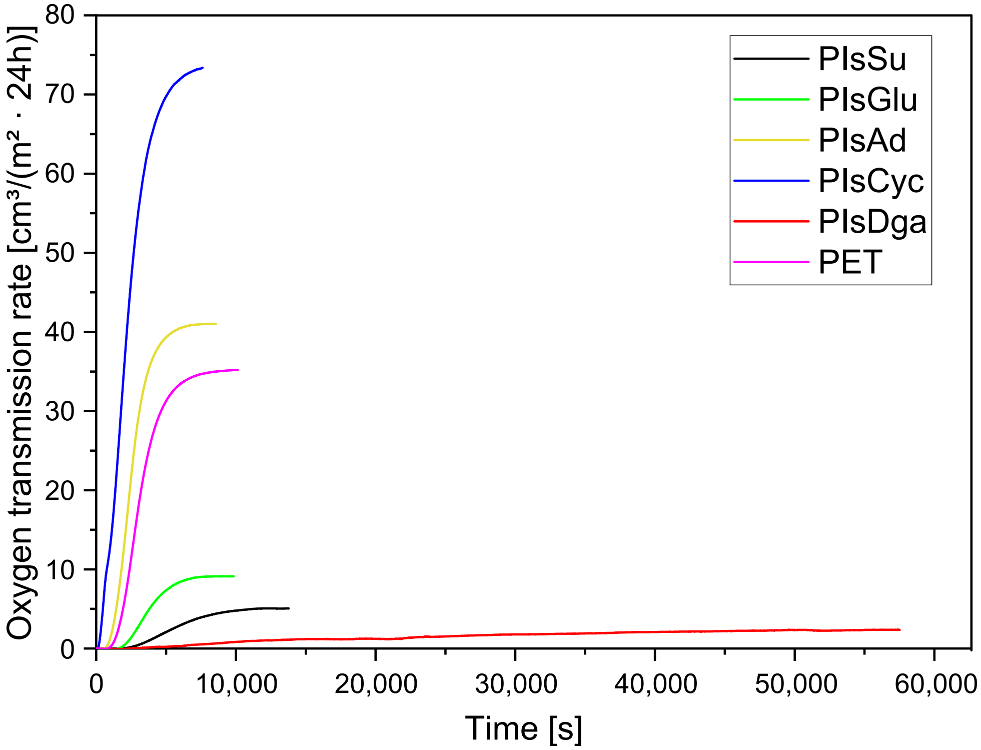

Supplementary Figure 47. Oxygen transmission rate curves of isosorbide-based polyesters and poly(ethylene terephthalate) (PET) recorded at 30 °C and 50% humidity.

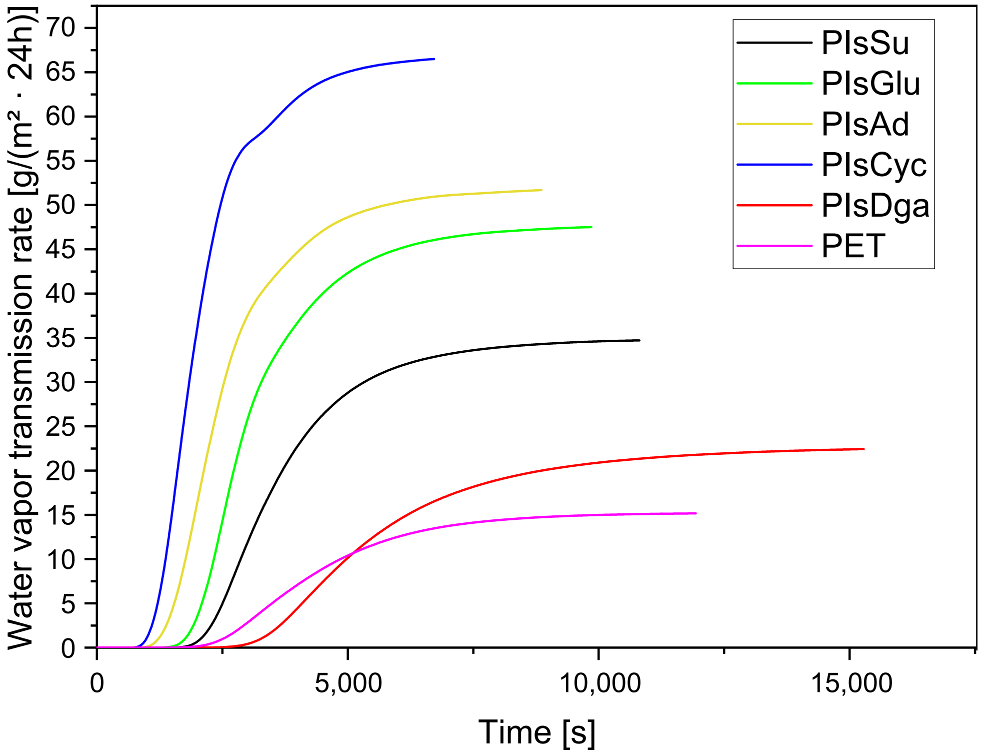

Supplementary Figure 48. Water vapour transmission curves of isosorbide-based polyesters and PET recorded at 38 °C and 90% humidity. The measurement of PIsAd was conducted at 30 ° due to the low T_g of the material.

Stress-strain graphs of isosorbide-based polyesters

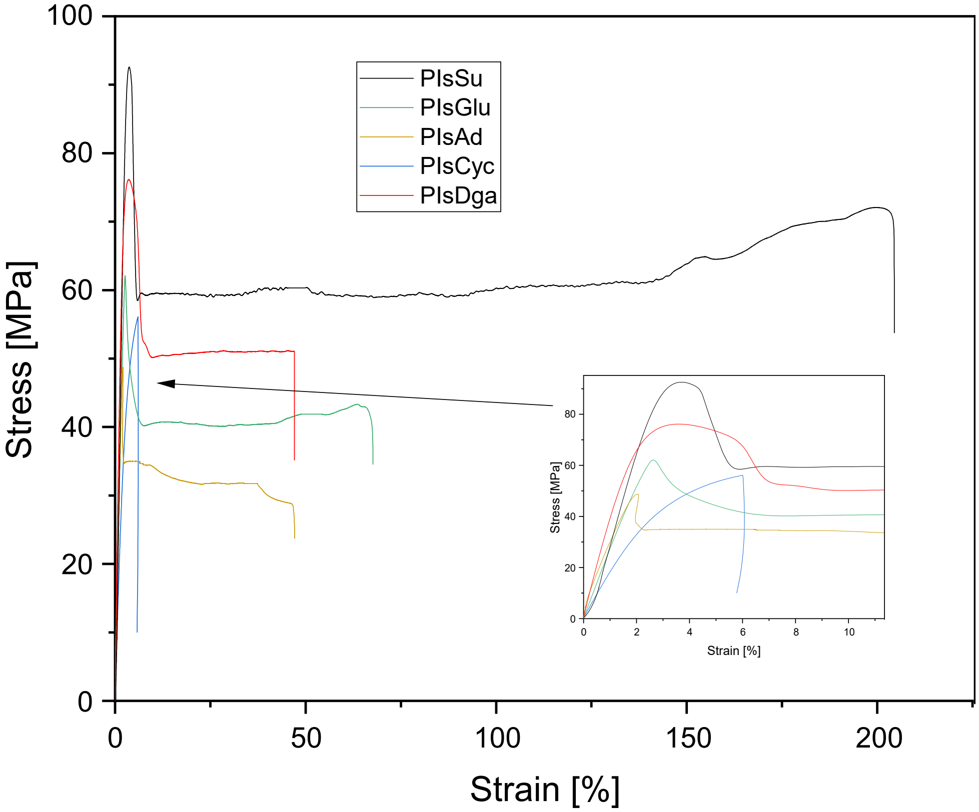

Supplementary Figure 49. Representative stress-strain plots of isosorbide-based polyesters. Extensions beyond 12 mm were measured after the Extensometer was removed from the sample. The graphs were manually combined to show the full extensions of samples. PIsAd samples consistently necked above the Extensometer, which is why their full extension was not measured completely by the Extensometer.

Comparison of 100 mL glass reactor with 2 L steel reactor

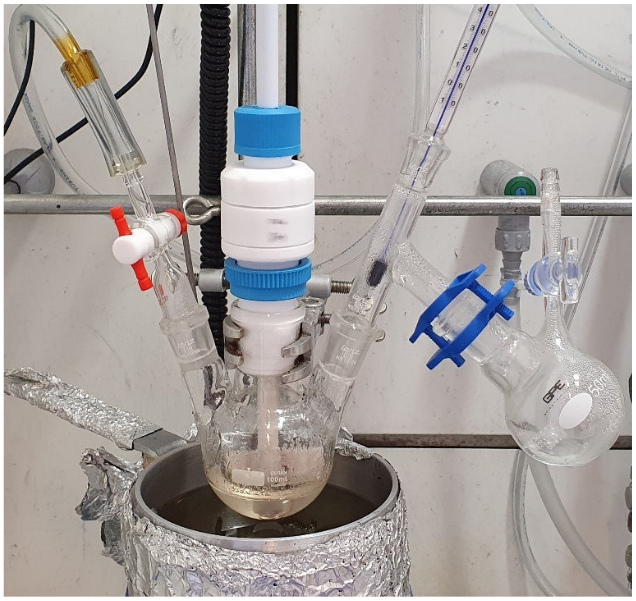

Supplementary Figure 50. Typical 100 mL glass reactor setup.

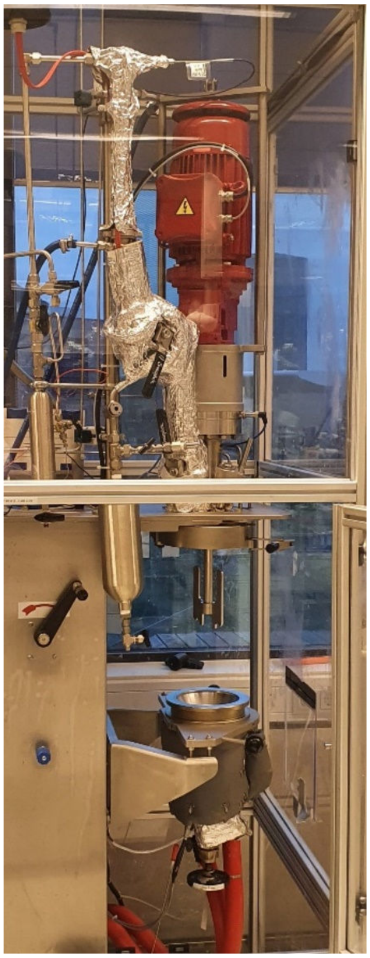

Supplementary Figure 51. 2 L stainless steel reactor used for poly(isosorbide succinate) upscaling experiments.

Collection of volatiles during esterification of poly(isosorbide succinate) in 2 L autoclave

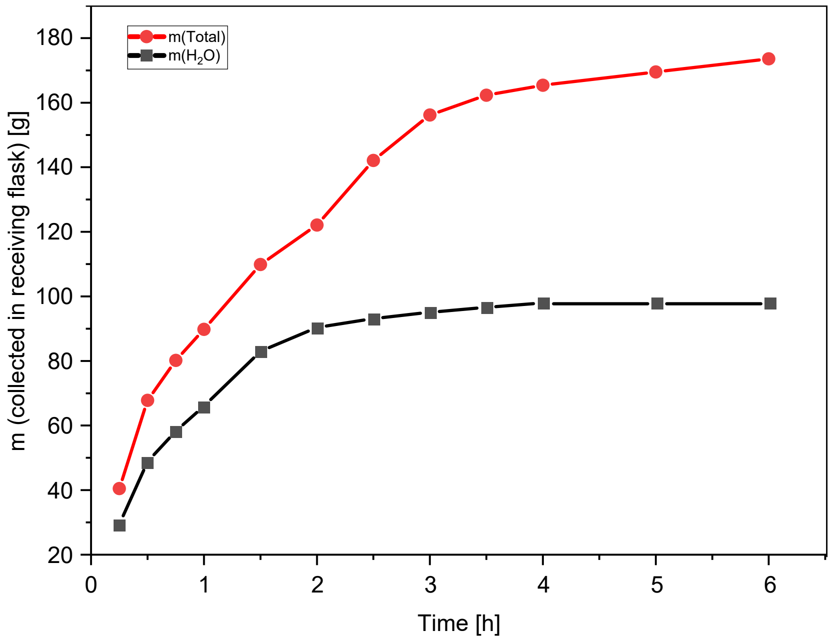

Supplementary Figure 52. Mass of volatiles collected during esterification between isosorbide, succinic acid and *p*-cresol in the 2 L autoclave. Volatiles were collected at the indicated times from both the short path receiving flask and the long path receiving flask (see Supplementary Figure 51). Typically a biphasic fraction was collected, consisting of H_2O and *p*-cresol. At later stages of esterification, only *p*-cresol was collected. The reaction temperature was increased from 220 °C to 240 °C after 1 h. The plateau value for m(H_2O) corresponds to 87.7% of theoretically expected H_2O.

Ring-opening hydration of isosorbide to 1,4-sorbitan

The ring-opening hydration of isosorbide to 1,4-sorbitan was only observed in experiments conducted in the 2 L autoclave. It can be explained by a lower surface of evaporation, which can cause an increased reflux of water during esterification. Indeed it was found that 1,4-sorbitan only forms during esterification. It might be avoided with a different reactor design, for example with a heated lid that decreases water reflux.

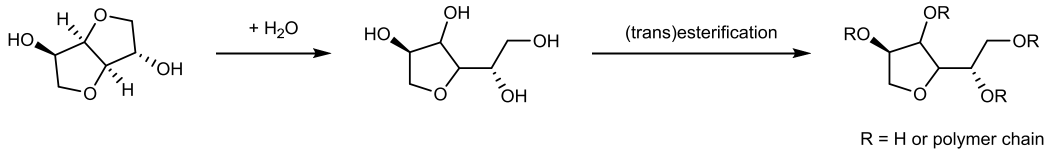

Supplementary Figure 53. Ring-opening hydration of isosorbide to 1,4-sorbitan and incorporation into the polymer chain.

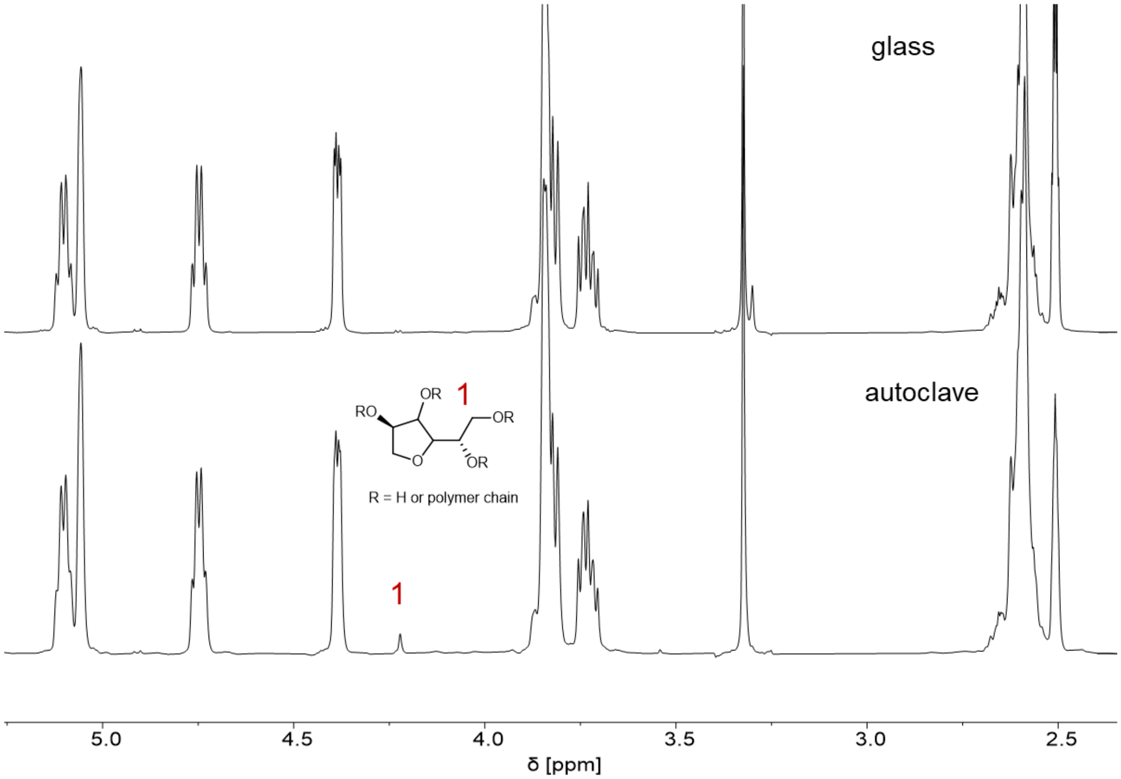

Supplementary Figure 54. ^{1}H NMR spectra (in DCM-d2) of poly(isosorbide succinate) synthesized in a 100 mL glass reactor (top) and in a 2 L stainless steel autoclave (bottom). The peak at 4.31 ppm corresponds to the primary alcohol of 1,4-sorbitan connected to the polymer chain, as attributed by Yoon *et al.*[3]. The other signals expected to appear can not be identified due to their small signal intensity, which is likely due to their secondary alcohol functionality. This, combined with the low overall amount of 1,4-sorbitan in the polymer chain (1.5 mol%), leads to low overall concentrations of protons of each species (connected or unconnected) in the polymer chain. The assigned peak 1 has a higher reactivity as a primary alcohol, which likely facilitates its complete esterification and thus enables quantification.

Calculation of mol% (respective total isosorbide units) of 1,4-sorbitan during esterification in 2 L autoclave

Due to the partial ring-opening hydration of isosorbide during esterification, small amounts of 1,4-sorbitan are formed. The ^{1}H chemical shift of 1,4-sorbitan in the oligomer chains is at 4.22 ppm, which overlaps with peaks of *endo*-OH monoesters of isosorbide and unreacted isosorbide (see Supplementary Figure 2 for assignments of relevant end groups after esterification).

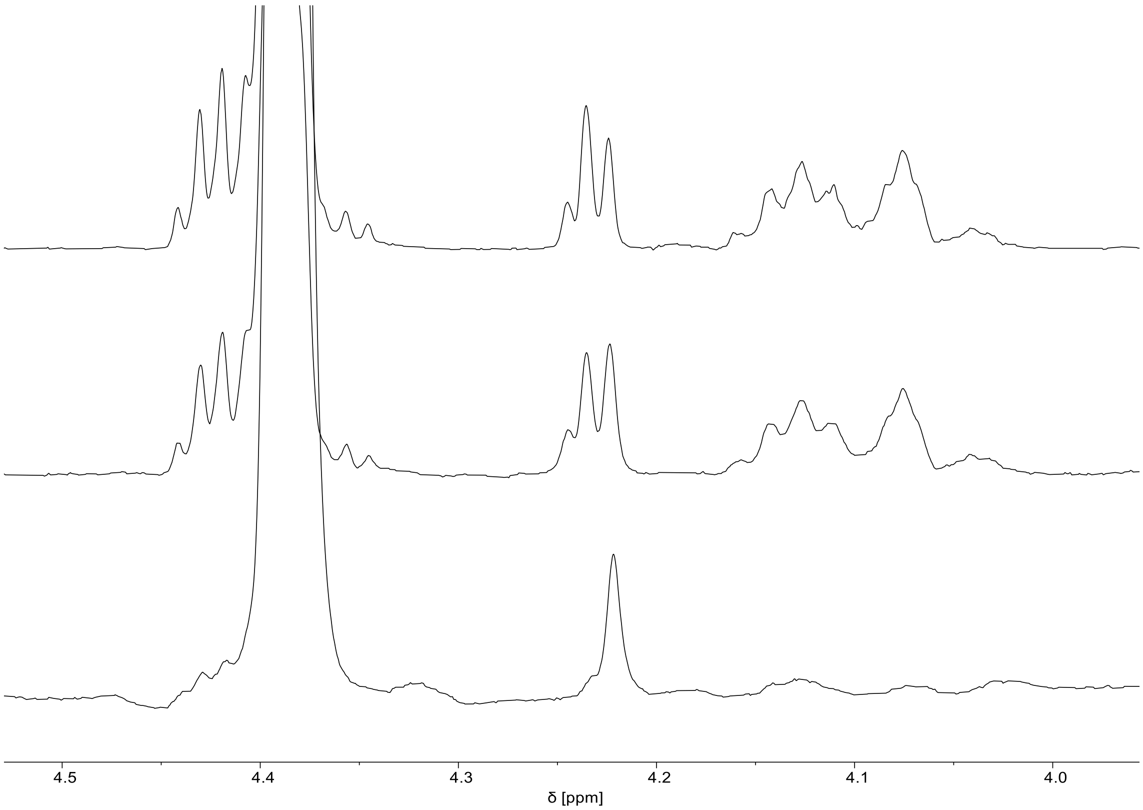

Supplementary Figure 55. ^{1}H NMR spectra of esterification mixture (after 5 h) in glass (top), in the 2 L autoclave (middle) and of the final product synthesized in the 2 L autoclave (bottom). The peak at 4.22 ppm indicates the presence of 1,4-sorbitan in the polymer chain.

The mol% of 1,4-sorbitan in the oligomer and polymer chain was calculated as follows:

$$mol\%(1{,}4-sorbitan) = \frac{\frac{A(4.22-4.27\ ppm)-A(endo-monoester, 4.67\ ppm)-A(unreacted\ IS, 4.03\ ppm)}{2}}{A(endo-monoester, 4.67\ ppm)+A(exo-monoester, 4.93\ ppm)+A\ (IS\ repeat\ unit, 4.74\ ppm)+A(unreacted\ IS, 4.03\ ppm)} * 100$$

The amount of 1,4-sorbitan did not change after esterification.

Temperature, torque and pressure evolution during poly(isosorbide succinate) synthesis in 2 L autoclave

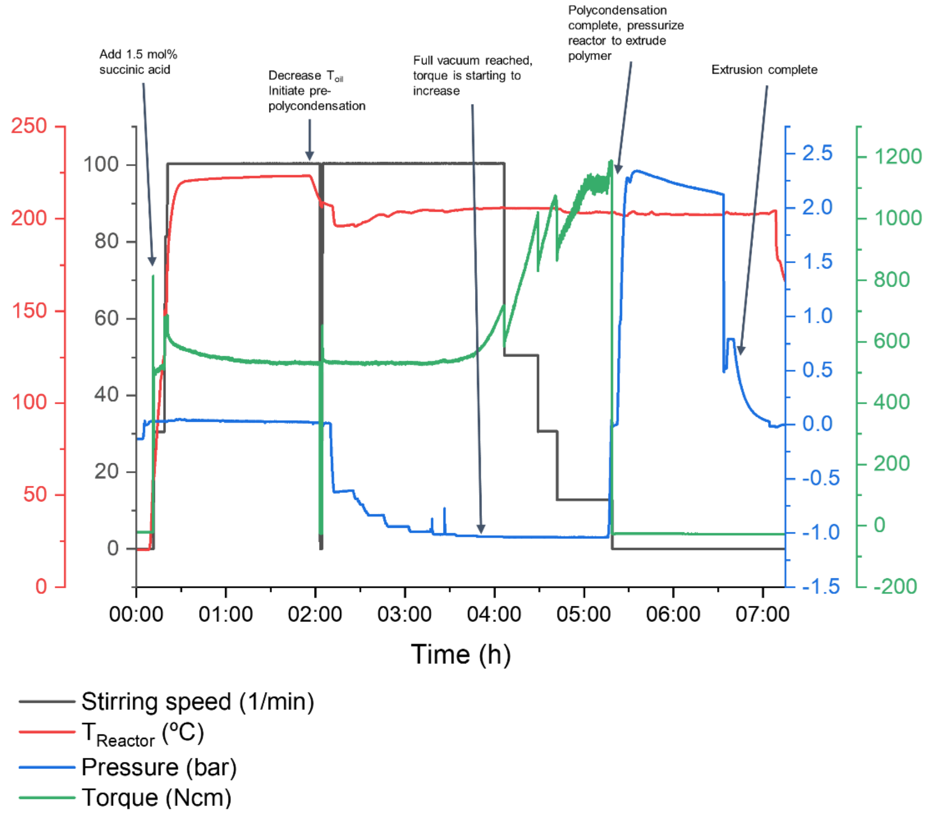

Supplementary Figure 56. Reactor parameters during the synthesis of poly(isosorbide succinate) in a 2 L autoclave. The reaction after addition of 1.5 mol% succinic acid (to compensate for the ring opening reaction of isosorbide to 1,4-sorbitan), pre-polycondensation and polycondensation is depicted. No significant changes in reactor parameters can be seen during the initial esterification. For a plot of the volatiles collected during esterification, see Supplementary Figure 52.

Poly(isosorbide succinate) from 2 L autoclave

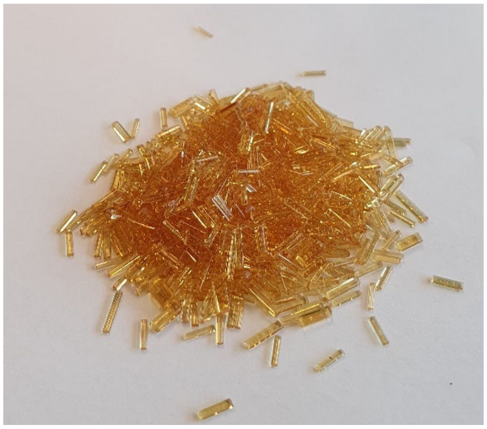

Supplementary Figure 57. Poly(isosorbide succinate) obtained after extrusion from a 2 L steel autoclave and chipping.

Soil burial degradability of poly(isosorbide succinate)

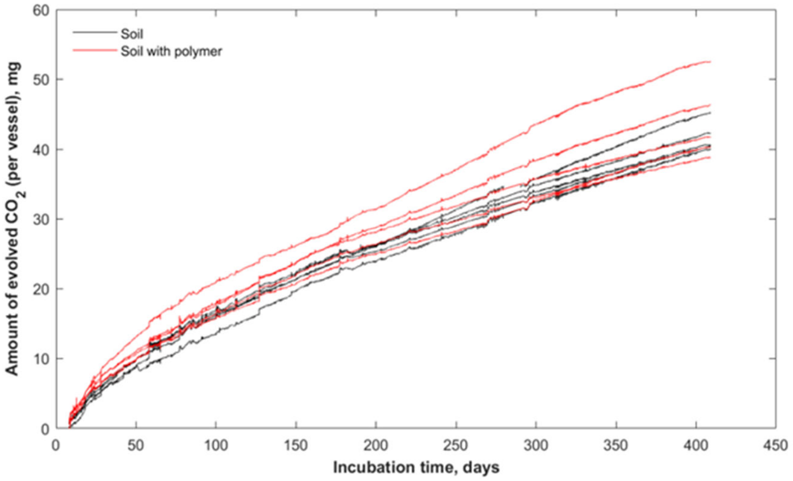

Supplementary Figure 58. Biodegradability of poly(isosorbide succinate) determined in soil burial experiments. No significantly increased evolution of CO_2 compared to blank vessels with only soil was observed after more than a year, which indicates no significant biodegradation (see Materials and Methods for exact conditions).

Hydrolytic degradability of poly(isosorbide succinate)

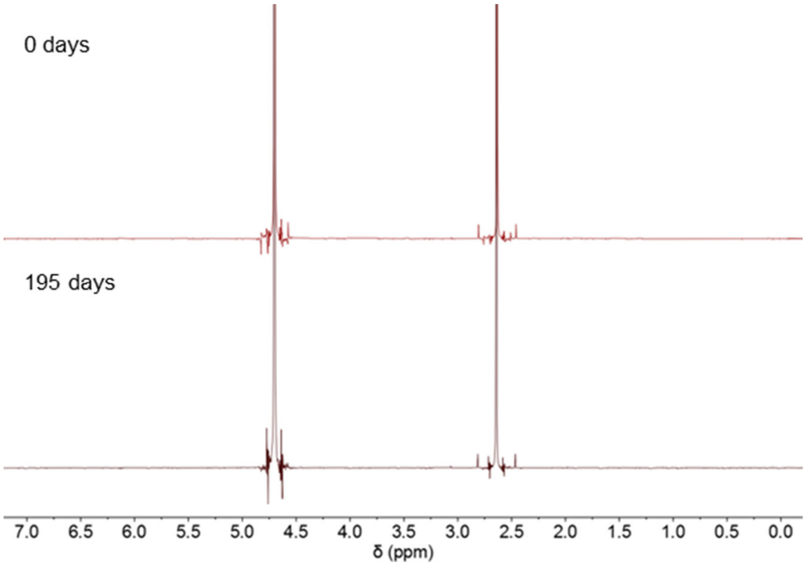

Supplementary Figure 59. Hydrolytic degradation of poly(isosorbide succinate) as observed at neutral pH in D_2O. Samples were grinded, sieved an weighed into an NMR tube with D_2O. No soluble compounds were found after 195 days at 25 °C. For exact sample preparation and measurement conditions, see Materials and Methods.

Supplementary Tables

Synthesis conditions for isosorbide-and isomannide-based polyesters

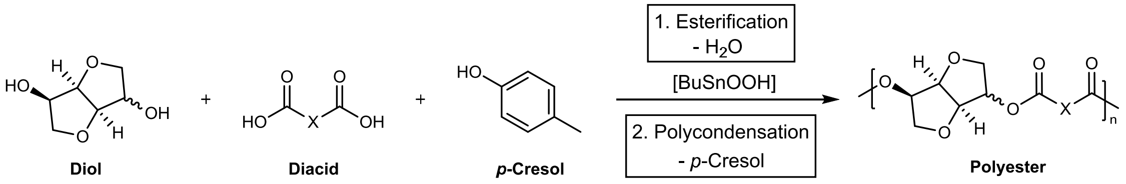

Supplementary Table 1. Synthesis conditions for isosorbide (IS)- and isomannide(IM)-based polyesters described in Table 5.

Diol	Diacid	Polyester	n (cat.) [mol%] [a]	Est. T [°C]	Est. t [h]	Polycond. T [°C]	Polycond. t [min] [b]
IS	Succinic acid	PIsSu [c]	0.1	240	5	220	60
IS	Glutaric acid	PIsGlu	0.2	240	8	220	140
IS	Adipic acid	PIsAd [d]	0.2	240	5	220	240
IS	1,4-Cyclohexanedicarboxylic acid	PIsCyc [e]	0.2	240-250	12	240-260	120
IS	Diglycolic acid	PIsDga [f]	0.1	200-240	6	200	215
IS	Thiodiglycolic acid	PIsThd [g]	0.1 [h]	200-220	8.5	200	125
IM	Succinic acid	PImSu	0.2	240	5.5	220	90
IM	Glutaric acid	PImGlu	0.2	240	8	220	210
IM	Adipic acid	PImAd [i]	0.2	240	7	220	180
IM	1,4-Cyclohexanedicarboxylic acid	PImCyc [j]	0.2	240-250	11	240-260	145
IM	Diglycolic acid	PImDga	0.1	200-240	7	200-240	210

Esterification was typically conducted until a steady state equilibrium was reached, which indicated full conversion of carboxylic acid end groups. Unless noted otherwise, reactions were conducted with a 1:1:1.5 molar ratio of diol:diacid:*p*-cresol, on a 180 mmol (of the respective diol) scale and a BuSnOOH catalyst. [a] Respective diacid. [b] Refers to the reaction time at full vacuum (<1 mbar). [c] Reaction was conducted with 60 mmol of diol. [d] A 2 mol% (respective isosorbide) excess of adipic acid was added due to decomposition of adipic acid to cyclopentanone during esterification. [e] Amount of trans 1,4-cyclohexanedicarboxylic acid in product: 58.2%. Before reaction: 22.8%. Isomerization during reaction was also described by Yoon *et al.*[3]. [f] Lower thermal stability of diglycolic acid required lower initial esterification T and lower polycondensation T. [g] A 0.7 mol% (respective isosorbide) excess of thiodiglycolic acid was used due to decomposition of the monomer. The final product was brittle. [h] $Ti(OBu)_4$ was used as a catalyst, BuSnOOH did not catalyse the polycondensation reaction sufficiently. The catalyst was added in two portions (before and after esterification) to compensate for the hydrolytic sensitivity of Ti-alkoxides. [i] A 0.7 mol% (respective isomannide) excess of adipic acid was added due to decomposition of adipic acid to cyclopentanone during esterification. [j] Amount of trans 1,4-cyclohexanedicarboxylic acid in product: 60.6%. Before reaction: 22.8%.

Alcohol to ester end group ratios and mol% of unreacted diol monomers of all polyester compositions after esterification

Supplementary Table 2. Alcohol to ester end group ratios and mol% of unreacted diol monomers of all polyester compositions after esterification. Values were determined as described on page 6 of the Supplementary Information. Typically esterifications were conducted until both values reached plateau values.

Polymer	Alcohol to ester end group ratio	Mol% unreacted diol (IS or IM) respective total diol units
Poly(isosorbide succinate) (PIsSu)	0.85	2.1
Poly(isosorbide glutarate) (PIsGlu)	0.85	1.7
Poly(isosorbide adipate) (PIsAd)	0.89	1.7
Poly(isosorbide-1,4-cyclohexanedicarboxylate) (PIsCyc)	0.81	1.5
Poly(isosorbide diglycolate) (PIsDga)	0.95	1.6
Poly(isosorbide thiodiglycolate) (PIsThd)	0.96	2.0
Poly(isomannide succinate) (PImSu)	0.79	2.3
Poly(isomannide glutarate) (PImGlu)	0.84	2.4
Poly(isomannide adipate) (PImAd)	0.83	2.3
Poly(isomannide-1,4-cyclohexanedicarboxylate) (PImCyc)	0.81	2.5
Poly(isomannide diglycolate) (PImDga)	0.92	2.4

$T_{5\%d}$ values of polyester compositions

Supplementary Table 3. $T_{5\%d}$ values of the synthesized polyesters.

Polymer	$T_{5\%d}$ [°C]
Poly(isosorbide succinate) (PIsSu)	367
Poly(isosorbide glutarate) (PIsGlu)	371
Poly(isosorbide adipate) (PIsAd)	347
Poly(isosorbide-1,4-cyclohexanedicarboxylate) (PIsCyc)	390
Poly(isosorbide diglycolate) (PIsDga)	363
Poly(isosorbide thiodiglycolate) (PIsThd)	337
Poly(isomannide succinate) (PImSu)	371
Poly(isomannide glutarate) (PImGlu)	388
Poly(isomannide adipate) (PImAd)	345
Poly(isomannide-1,4-cyclohexanedicarboxylate) (PImCyc)	396
Poly(isomannide diglycolate) (PImDga)	362

Conditions used for compression moulding of polymer films and average thicknesses

Supplementary Table 4. Hot press temperatures used to prepare polymer films and average films thicknesses

Polymer	T (thermal press) [°C]	Average thickness [µm]
PIsSu glass	190	110
PIsSu autoclave	190	110
PIsGlu	160	110
PIsAd	120	100
PIsCyc	260	100
PIsDga	190	100
PET	270	100

Conditions used for injection moulding of polymer tensile bars

Supplementary Table 5. Conditions used for injection moulding of polymer tensile bars.

Polymer	T (cylinder) [°C]	T (mould) [°C]	Residence time in heated cylinder [s]
PIsSu glass	180-190	50	60-90
PIsSu autoclave	180-190	50	60-90
PIsGlu	150	35	60-90
PIsAd	90-100	25	120
PIsCyc	255	60	180-210
PIsDga	145-150	50	120-150

Influence of injection moulding on PIsSu molecular weight

Supplementary Table 6. Molecular weight decrease of PIsSu before and after injection moulding. The polymer was dried at 40 °C for 24 h prior to injection moulding. Residence time in heated cylinder ($T_{cylinder}$ = 185 °C) before injection: 90 seconds.

Processing stage	M_n [g/mol]	PDI
Before inj. moulding	35.3	2.2
After inj. moulding	32.2	2.2

Comparison of mechanical properties of PIsSu synthesized in glass reactor and 2 L autoclave

Supplementary Table 7. Comparison of mechanical properties of PIsSu synthesized in glass reactor and 2 L autoclave. The small differences can be explained by the presence of 1.5 mol% 1,4-sorbitan in the polymer chain of PIsSu samples synthesized in the 2 L autoclave.

Reactor type	M_n [g/mol]	PDI	Young's Modulus [MPa]	Ultimate tensile strength [MPa]	Elongation at break [%]
Glass	35.7	2.1	3,696 ±169	78.9 ± 5.0	175.3 ± 4.5
Autoclave	35.3	2.2	3,760 ± 29	88.2 ± 3.3	196.9 ± 19.8

Comparison of barrier properties of PIsSu synthesized in glass reactor and 2 L autoclave

Supplementary Table 8. Comparison of barrier properties of PIsSu synthesized in glass reactor and 2 L autoclave.

Reactor type	M_n [g/mol]	PDI	Film thickness [µm]	Oxygen permeability [$mm \cdot cm^3/m^2 \cdot day^1 \cdot bar^1$]	Water vapor permeability [$mm \cdot cm^3/m^2 \cdot day^1 \cdot bar^1$]
Glass	38.2	2.4	110	0.79	3.83
Autoclave	35.3	2.2	110	0.56	3.82

References

1. Noordover, B. A. J. *et al.* Co- and Terpolyesters Based on Isosorbide and Succinic Acid for Coating Applications: Synthesis and Characterization. *Biomacromolecules* **7**, 3406–3416 (2006).

2. Okada, M., Okada, Y. & Aoi, K. Synthesis and degradabilities of polyesters from 1,4:3,6-dianhydrohexitols and aliphatic dicarboxylic acids. *Journal of Polymer Science Part A: Polymer Chemistry* **33**, 2813–2820 (1995).

3. Yoon, W. J. *et al.* Advanced Polymerization and Properties of Biobased High Tg polyester of Isosorbide and 1,4-Cyclohexanedicarboxylic Acid through in Situ Acetylation. *Macromolecules* **46**, 2930–2940 (2013).
